# IHMValidation: Assessment of Integrative Structure Models Deposited to the Protein Data Bank^☆^

**DOI:** 10.1016/j.jmb.2025.169598

**Published:** 2025-12-18

**Authors:** Arthur O. Zalevsky, Brinda Vallat, Benjamin M. Webb, Hongsuda Tangmunarunkit, Monica R. Sekharan, Aref Shafaeibejestan, Sai Ganesan, Jared Sagendorf, Cy M. Jeffries, Jill Trewhella, Andrea Graziadei, Juan Antonio Vizcaíno, Alexander Leitner, Juri Rappsilber, Ezra Peisach, Justin W. Flatt, Jasmine Y. Young, Kartik Majila, Shruthi Viswanath, Carl Kesselman, Jeffrey C. Hoch, Genji Kurisu, Kyle L. Morris, Sameer Velankar, Helen M. Berman, Stephen K. Burley, Andrej Sali

**Affiliations:** 1 - Research Collaboratory for Structural Bioinformatics Protein Data Bank, Department of Bioengineering and Therapeutic Sciences, the Quantitative Biosciences Institute (QBI), and the Department of Pharmaceutical Chemistry, University of California, San Francisco, San Francisco, CA 94157, USA; 2 - Research Collaboratory for Structural Bioinformatics Protein Data Bank and the Institute for Quantitative Biomedicine, Rutgers, The State University of New Jersey, Piscataway, NJ 08854, USA; 3 - Rutgers Cancer Institute, Rutgers, The State University of New Jersey, New Brunswick, NJ 08901, USA; 4 - Information Sciences Institute, Viterbi School of Engineering, University of Southern California, Los Angeles, CA, USA; 5 - European Molecular Biology Laboratory, Hamburg Unit, c/o Deutsches Elektronen-Synchrotron, Notkestraße 85, 22607 Hamburg, Germany; 6 - Ilse Katz Institute for Nanoscale Science and Technology, Ben-Gurion University of the Negev, Be’er Sheva 8410501, Israel; 7 - School of Life and Environmental Sciences, University of Sydney, NSW 2006, Australia; 8 - Department of Chemistry, University of Utah, Salt Lake City, UT 84112, USA; 9 - Human Technopole, Via Rita Levi Montalcini 1, 20157 Milano, Italy; 10 - European Molecular Biology Laboratory, European Bioinformatics Institute (EMBL-EBI), Wellcome Trust, Genome Campus, Hinxton, Cambridge CB10 1SD, UK; 11 - Institute of Molecular Systems Biology, Department of Biology, ETH Zürich, 8093 Zurich, Switzerland; 12 - Technische Universität Berlin, Chair of Bioanalytics, 10623 Berlin, Germany; 13 - Si-M/“”Der Simulierte Mensch“”, a Science Framework of Technische Universität Berlin and Charité – Universitätsmedizin Berlin, Berlin, Germany; 14 - National Center for Biological Sciences, Tata Institute of Fundamental Research, Bangalore, Karnataka 560065, India; 15 - Biological Magnetic Resonance Data Bank, Department of Molecular Biology and Biophysics, University of Connecticut, Farmington, CT 06030-3305, USA; 16 - Protein Data Bank Japan, Institute for Protein Research, Osaka University, Suita, Osaka 565-0871, Japan; 17 - Electron Microscopy Data Bank, European Molecular Biology Laboratory, European Bioinformatics Institute, Hinxton, Cambridge CB10 1SD, UK; 18 - Protein Data Bank in Europe, European Molecular Biology Laboratory, European Bioinformatics Institute, Hinxton, Cambridge CB10 1SD, UK; 19 - Department of Chemistry and Chemical Biology, Rutgers, The State University of New Jersey, Piscataway, NJ 08854, USA; 20 - Department of Quantitative and Computational Biology, University of Southern California, Los Angeles, CA 90089, USA; 21 - Research Collaboratory for Structural Bioinformatics Protein Data Bank, San Diego Supercomputer Center, University of California, La Jolla, CA 92093, USA; 22 - Rutgers Artificial Intelligence and Data Science (RAD) Collaboratory, Rutgers, The State University of New Jersey, Piscataway, NJ 08854, USA

**Keywords:** integrative modeling, Protein Data Bank (PDB), Small-angle X-ray scattering (SAXS), crosslinking mass spectrometry (crosslinking-MS), three-dimensional cryo-electron microscopy (3DEM)

## Abstract

PDB-IHM is a branch of the Protein Data Bank (PDB), a Worldwide Protein Data Bank (wwPDB) Core Archive, that expands its scope by allowing for additional biomolecular structure representations and types of experimental information (*i.e.*, integrative/hybrid structure models). As of October 2025, PDB-IHM contained 374 entries, benefitting from multi-scale and multi-state representations and 17 types of experimental data. These structure models are assigned PDB accession codes and are archived alongside other experimental structures in the PDB. Rigorous interpretation of a structure model requires assessment of underlying data quality, consistency with the input data, and estimates of positional uncertainty of its components. Herein, we present the IHMValidation pipeline (https://validate.pdb-ihm.org; https://github.com/salilab/IHMValidation) based on recommendations from the wwPDB Integrative Methods Task Force plus the small-angle scattering (SAS), chemical crosslinking mass spectrometry (crosslinking-MS), and cryo-electron microscopy and tomography (3DEM) communities. The IHMValidation report (available in both PDF and HTML formats) comprises six sections: (i) overview; (ii) model details; (iii) data quality assessments; (iv) local geometry assessments (*i.e.*, model quality); (v) fit of the model to the data used to generate it; and (vi) fit of the model to the data used for validation. Future expansions of the IHMValidation pipeline will: (i) reflect recommendations coming from additional experimental communities, including Förster resonance energy transfer (FRET) and hydrogen/deuterium exchange MS (HDX-MS); (ii) include other validation criteria, such as Bayesian likelihoods for the data; and (iii) represent estimates of structure model uncertainty based on the variation among alternative models satisfying input data.

## Graphical Abstract

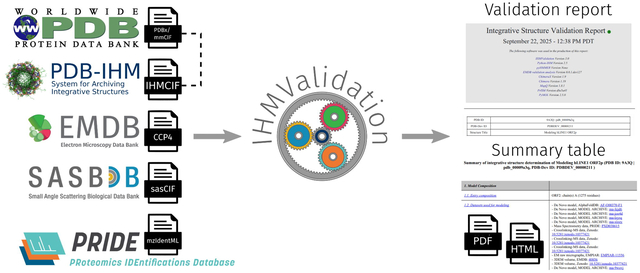

## Introduction

Protein Data Bank (PDB) [1–3] is the single global archive of three-dimensional (3D) structure models of biological macromolecules and their complexes determined by X-ray crystallography (MX) (82% of holdings as of October 2025), three-dimensional cryo-electron microscopy (3DEM, 12%), nuclear magnetic resonance (NMR) spectroscopy (6%), and other techniques (<1%), totalling more than 243,000 entries. The PDB is one of three jointly managed Worldwide Protein Data Bank (wwPDB) [4] Core Archives, the other two being the Electron Microscopy Data Bank (EMDB) [5] and the Biological Magnetic Resonance Data Bank (BMRB) [6]. Recently, the scope of the PDB was expanded by PDB-IHM (PDB Integrative and Hybrid Methods; previously PDB-Dev [7,8]) [9]. PDB-IHM is a branch of the PDB that allows for additional biomolecular structure representations and types of experimental information, supporting deposition, validation, archival, and dissemination of integrative/hybrid structure models (hereafter abbreviated as “integrative structure models”).

Integrative modeling aims to maximize accuracy, precision, completeness, and explanatory power of a 3D structural model by combining multiple types of input information: experimental data (*e.g*., NMR data, chemical crosslinks), physical theories (*e.g*., stereochemistry quantified by a molecular mechanics force field), statistical preferences (*e.g*., atomic statistical potentials extracted from known structures), and previous structural models (*e.g*., structural models of complex subunits). For example, an integrative structure model is often computed using a 3DEM map, crosslinks determined by chemical crosslinking mass spectrometry (crosslinking-MS), and stereochemistry restraints. Depending on the resolution of the data and the scale of the modeled system, a modeler may choose to compute a model of any one of several types, including coarse-grained, multi-scaled, multi-state, and ordered-state [10]. To annotate new experimental data types and model representations, the wwPDB PDB-IHM team developed the IHM extension of the PDBx/mmCIF dictionary, called IHMCIF [11].

**Figure 1.**
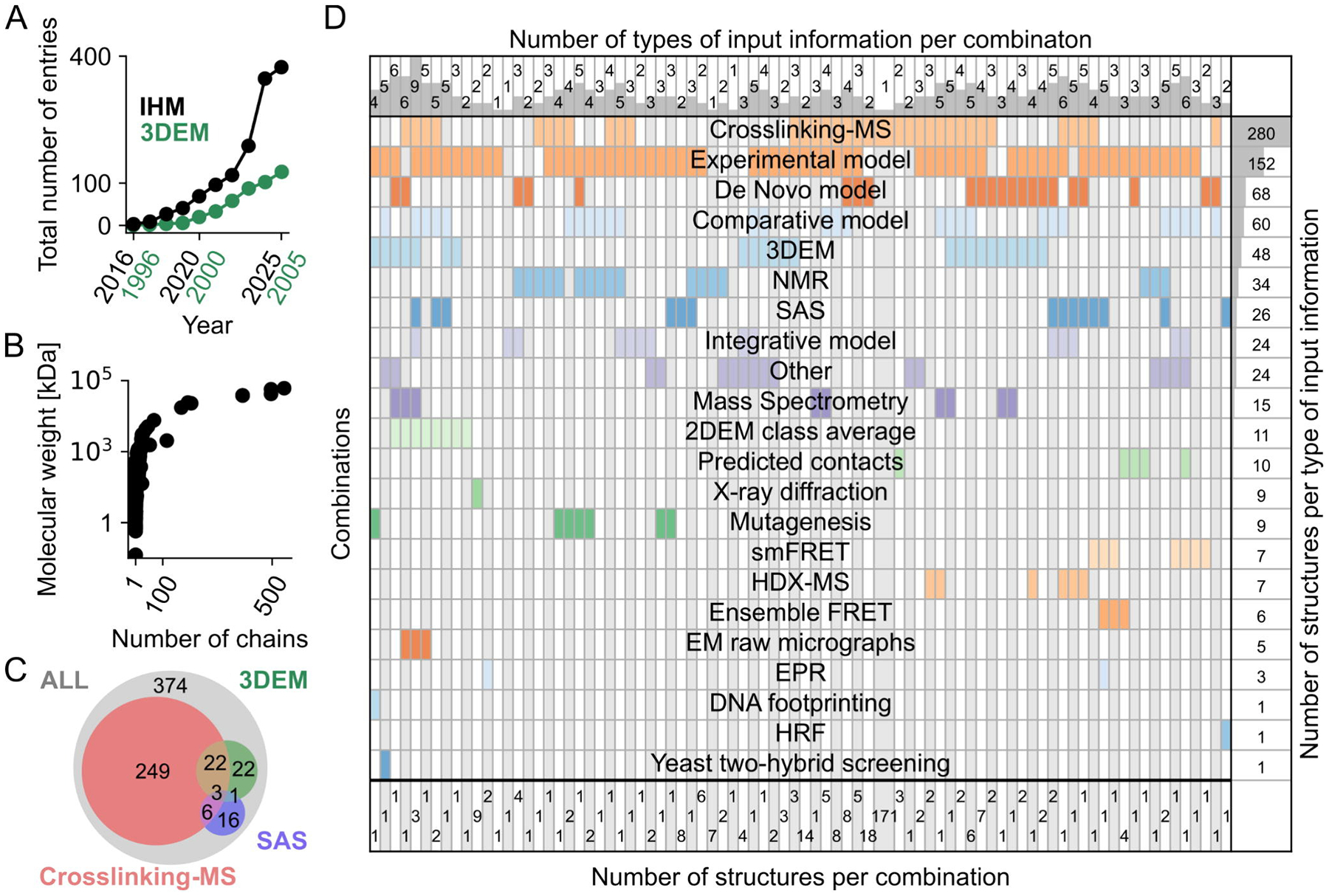
PDB-IHM contents. **(A)** Growth in the number of integrative structure models in PDB-IHM (black filled circles). For comparison, the initial growth in the number of 3DEM structures between 1996 and 2005 is also shown (filled green circles). We excluded the period from 1991 to 1995, because only one 3DEM structure was deposited in that timeframe. **(B)** Molecular weight of the PDB-IHM structure *versus* number of polymer chains. **(C)** Venn diagram of PDB-IHM entries coming from 3DEM, SAS, and crosslinking-MS data; collectively, they represent 85% of all released entries. **(D)** Experimental methods and structural data sources used to generate PDB-IHM structures are presented in a matrix. Each row corresponds to a source of input information, including crosslinking mass spectrometry (crosslinking-MS), various forms of electron microscopy (EM), nuclear magnetic resonance (NMR) spectroscopy, small-angle scattering (SAS), single-molecule and ensemble Förster resonance energy transfer (smFRET, Ensemble FRET), hydrogen–deuterium exchange MS (HDX-MS), electron paramagnetic resonance (EPR), and hydroxyl radical footprinting (HRF). The number of entries with a given type of input information is plotted on the right. Each column represents a particular combination of input information. Counts in the top section indicate the number of sources of input information in the column; at the bottom, the number of entries with a given combination of sources of input information.

As of October 2025, PDB-IHM contained 374 publicly released entries (Figure 1A). Most (~74%) of these entries are multi-chain (homo- and hetero-oligomeric) structures; 56% of the entries have a molecular weight greater than 80 kDa, with an overall range from 5.8 kDa to 3.8 MDa (Figure 1B). This distribution is similar to that for the main PDB archive.

PDB-IHM entries vary in the types of experimental data on which they are based. Unlike the main PDB archive, which primarily consists of structures determined based on the MX, 3DEM, and NMR data, the current PDB-IHM entries rely on 17 different types of experiments, including various forms of electron microscopy (EM), crosslinking-MS, small-angle scattering (SAS), NMR spectroscopy, mutagenesis, single-molecule Förster resonance energy transfer (smFRET), hydrogen–deuterium exchange MS (HDX-MS), X-ray diffraction, electron paramagnetic resonance (EPR), DNA footprinting, and hydroxyl radical footprinting (HRF). Overall, 83 unique combinations of these input data types were used to compute the current PDB-IHM structure models (Figure 1D).

Integrative modeling generally benefits from previously determined starting structures for individual components of the modeled system [10,12,13]. Half of the PDB-IHM structure models were generated using at least one starting structure; conformations of these structures can be fixed or flexible during integrative modeling. Starting structures can be determined by any structural biology method, including experimental methods (*e.g*., X-ray crystallography, NMR spectroscopy, 3DEM) and computational methods (*e.g.*, comparative modeling, *de novo* structure prediction [14]). Conveniently, the atomic coordinates of these starting structures can often be downloaded from the PDB [2], PDB-IHM [9], Model Archive [15], or other public domain repositories. A substantial portion of PDB-IHM entries (43%) contain at least one polymer component extracted from an experimental structure archived in PDB (~41%) or a PDB-IHM integrative structure model (~6%). Similarly, ~29% of the PDB-IHM entries also contain at least one component polymer structure computed by comparative modeling (~16%), using software tools such as MODELER [16], SwissModel [17], and Rosetta [18], or *de novo* structure prediction (~18%), using programs such as RoseTTAFold [19] and AlphaFold2/3 [20,21]. We anticipate a rapid increase in the use of *de novo* models due to their availability, coverage, and accuracy. This trend has already been observed with recent PDB-IHM depositions (74% of entries released in 2025).

Rigorous interpretation of a structure requires assessment of underlying data quality, consistency with the input data, and estimates of positional uncertainty of the generated Cartesian coordinates. Herein, we present the IHMValidation pipeline based on recommendations from the wwPDB Integrative/Hybrid Methods Task Force (IHMtf) [22,23] plus the SAS [24,25], crosslinking-MS [26,27], and 3DEM [28] communities. We prioritized implementing support for SAS, crosslinking-MS, and 3DEM data because they accounted for 85% of the PDB-IHM entries (Figure 1C).

## Results and Discussion

### Validation report

Inspired by previous efforts on structural validation [24,28–30], we organized information about each structure model, including experimental and structural data used for structure model generation, model representation, details of the modeling protocol, and validation metrics, into two key documents: the full validation report and a summary table (Supplementary Files 1 and 2). The full validation report includes extended statistics and detailed plots. The summary table is a concise description of the full validation report, without graphical displays. The IHMValidation pipeline generates human-readable reports in both PDF and HTML formats.

The validation report is organized into six sections: (i) overview; (ii) model details; (iii) data quality assessments; (iv) local geometry assessments (*i.e.*, model quality); (v) fit of the model to the data used to generate it; and (vi) fit of the model to the data used for validation. Each section contains validation metrics presented as tables with numerical values and plots. Selection of metrics depends on the type of structural (atomic, coarse-grained, multi-scale) and the type of experimental data used to generate the integrative structure model, following recommendations from the IHMtf [22,23] and data-generating communities [24–28]. Next, we describe each section.

### Overview

**Figure 2.**
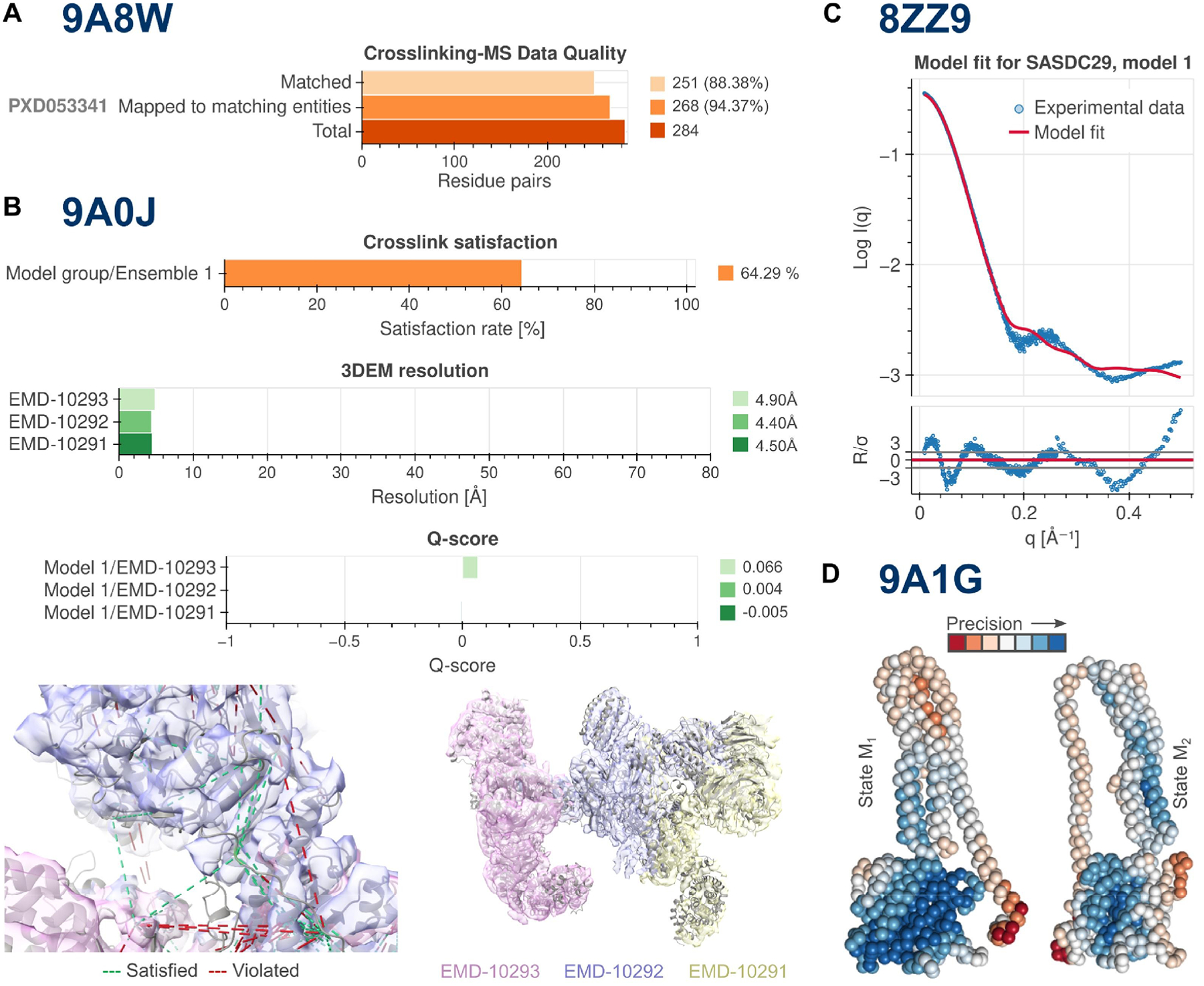
Typical issues highlighted by the IHMValidation reports. **(A)** The number of crosslinks used for modeling versus those present in the original dataset. (N.B.: Crosslinking-MS experiments can identify thousands of proteins, of which only a fraction are used for generating a particular PDB-IHM entry). For 9A8W, 268 of 284 crosslinks reported in the PRIDE dataset (PXD053341) were identified between proteins of interest, and only 251 of them were used for integrative modeling due to truncations of flexible regions [67]. **(B)** Fit of structural model to crosslinking-MS and 3DEM data. Integrative structure models are often computed using low-to-medium resolution data (*e.g*., crosslinking-MS and mid-resolution cryo-EM maps). (N.B.: Lower resolution 3DEM maps (~5 Å) typically exhibit average per residue Q-scores of ~0.3 [68]). Integrative modeling of 9A0J [69] utilized three different 3DEM maps (EMDB IDs: EMD-10291, EMD-10292, EMD-10293) to generate a more complete model that covers as many residues as possible. Spatial restraints derived from crosslinking-MS data provided complementary information about the relative positions of subunits across the maps. **(C)** Fit of structure to SAS data. At first glance, the agreement between the structure 8ZZ9 and the SAS data (SASBDB ID SASDC29) is unacceptably low (*χ*^2^ ~ 25). However, the authors rationalized the differences in the high-q region by the intrinsic dynamics of the protein, which was confirmed using NMR spectroscopy [70]. **(D)** Regions of low-(red) and high-(blue) local precision in the two collections of multi-state models of human guanylate-binding protein 1 (hGBP-1, 9A1G), annotated and visualized using PrISM. Two deposited collections of models (100 and 106 individual models in states M_1_ and M_2_, respectively) with substantially different representative structures for each state and noticeable differences in regions of high and low precision are shown [71].

This section provides an “executive” summary of the structure content and key quality indicators. The summary indicates serious problems with the structure, if any (Figure 2). The section contains plots of key metrics, divided into four categories, one for each validation criterion: (i) data quality assessments (Figure 2A, B); (ii) local geometry assessments (*i.e.*, model quality); (iii) fit of the model to the data used to generate it (Figure 2B, C) and (iv) fit of the model to the data used for validation.

### Model details

This section contains tables with detailed information about (i) the entities present in the structure (*e.g.*, biopolymers and ligands) and their representation (*i.e.*, atomic, coarse-grained, multi-scale, and multi-state); (ii) a description and links to datasets for computing the structure; and (iii) a summary of the modeling methodology (Supplementary File 1).

### Quality of data and fit of integrative structure models to data

Annotations can help depositors, biocurators, and data consumers identify problems with the underlying data, including artifacts resulting from experiments and data conversions into standardized formats.

With the data quality assessment in hand, we proceed to assess the integrative model against the data. IHMValidation distinguishes between two types of assessments. For both types, we use the same criteria to quantify the model-data match. First, the model is assessed against the data used to generate it. Ideally, the model should satisfy all of the input data within the specified data precision. Second, a model can be assessed with respect to holdout data (*i.e.*, data not used to compute the model), whether reported in the current study or previously published. Large deviation(s) of a model from holdout data can reveal structure overfitting [31,32], particularly when it is generated from a sparse dataset. Currently, we report assessments based on data used to generate the integrative structure model, while holdout assessment is planned for the next IHMValidation release.

### Quality of SAS data and fit of integrative structure models to data

Small-angle scattering (SAS) of X-rays (SAXS) or neutrons (SANS) by macromolecules in solution enables the characterization of their size and shape. In addition, as the SAS measurement yields the time and ensemble average of the molecules present in the solution, it provides information on structural heterogeneity relating to conformational or oligomeric state(s) present in the sample [33–36]. One advantage of SAS experiments is that the characterized system is in solution. While *in situ* measurements are sometimes feasible, most studies involve samples purified to high purity. SAS data can be useful for integrative modeling, especially for large and/or flexible macromolecules that may be difficult to study using other structural techniques alone. For example, the atom-pair distance distribution function (PDDF) computed from SAS data can be used as a spatial restraint for integrative structure modeling and/or to validate the resulting models [37–39].

The SAS portion of an IHMValidation report adheres to the wwPDB SAS Validation Task Force (SASvtf) recommendations [24]. PDB-IHM depositors are required to submit their SAS data to SASBDB [40], which serves as the source of SAS data and quality metrics in sasCIF format [41] as recommended by the SASvtf [24].

For data quality assessment the scattering profile is presented with data quality metrics, including Guinier analysis for the evaluation of the radius of gyration, *R*_*g*_, PDDF analysis for *R*_*g*_ and maximum particle dimension estimates (*D*_*max*_) as well as associated plots, *e.g*., Porod-Debye and Kratky plots that provide additional qualitative information relating to particle compactness, flatness, flexibility or extensibility (Supplementary Table 1A). PDB-IHM model metrics based on SAS data (Supplementary Table 1B) include plots of SAS profiles calculated for structures and their goodness-of-fit to experimental data in the form of the reduced *χ*^2^ with its associated error-weighted residual difference plot between model and experiment (Figure 2C), and *p*-values from the Correlation Map (CorMap) test.

### Quality of crosslinking-MS data and fit of integrative structure models to data

Crosslinking-MS can identify pairs of spatially proximal residues in a protein or protein complex [42]. After the sample is exposed to a chemical crosslinker, it is proteolytically digested (usually with trypsin), followed by detecting crosslinked pairs of residues by MS. These pairs can be converted into spatial restraints for integrative modeling and/or for validation of the resulting models [43]. The development of crosslinking reagents of varying length and chemical reactivity enables the study of compositionally and structurally homogeneous and heterogeneous protein complexes *in vitro* and *in situ* [44,45]. Moreover, the approach can also be applied to nucleic acids and their complexes with proteins [46].

The crosslinking-MS community is in the process of establishing its data standards [26,27], including protocols for validating crosslinking-MS data and structures. We propose here a set of metrics and visualization approaches to facilitate further discussion about community standards for validating this type of data, in coordination with members of the crosslinking-MS [27] community and the PRIDE database for proteomics data, including crosslinking-MS [47].

In the IHMValidation report, we first note the numbers of reported and utilized crosslinks. Crosslinking-MS data are retrieved from the PRIDE database *via* the Crosslinking-API or directly from a data file in the open standard mzIdentML format (version 1.2 or 1.3) [48]. We then compare proteins reported in the <DBSequence> element in mzIdentML with those reported in the IHMCIF file and match the corresponding cross-links. Percentages of crosslinks present in the IHMCIF file with respect to the crosslinking-MS data in the mzIdentML file are reported. Low percentage values indicate a potential problem with the omission of data, although sometimes only a fraction of crosslinks from an *in situ* or *in vivo* dataset is relevant for the current molecular system.

There is currently no generally accepted approach for performing a goodness-of-fit assessment between a 3D structural model and crosslinking-MS data. Widely used metrics/strategies include: (i) Euclidean or surface-based distances [49]; (ii) mapping of crosslinks onto cross-linked functional groups or Cα atoms [50]; (iii) cross-link satisfaction by a single conformation or a collection of conformations [51]; and (iv) continuous probabilistic scores as opposed to sharp cutoffs [52].

IHMValidation compares the structural model against the crosslink restraints used for integrative modeling as submitted by depositors. We provide a summary table listing restraints and corresponding user-provided thresholds. Next, we assess restraint satisfaction against the thresholds using Euclidean distances. The IHMValidation report attempts to account for modeling complexity during the assessment. For example, a single crosslink can be represented as a combination of lower and upper-bound distance restraints, or as a set of restraints between multiple instances of polymer entities, reflecting ambiguities in the crosslinking-MS dataset (*e.g.*, an intra-molecular crosslink *vs*. an inter-molecular homodimer crosslink).

Data quality metrics based on crosslinking-MS data include the total number of crosslinks in the dataset, the number of crosslinks matched to the structure, and the number of crosslinks matched to restraints (Supplementary Table 2A, Figure 2A). Integrative structure model metrics based on crosslinking-MS data include restraint types, distograms (*i.e.*, histogram plots of distances), and satisfaction rates (Supplementary Table 2B, Figure 2B).

### Quality of 3DEM data and fit of integrative structure models to data

3DEM, typically cryo-electron microscopy (cryoEM) and cryo-electron tomography (cryoET), is a powerful technique for studying 3D structures of biomolecules at low-to-medium-to-high resolution. CryoEM involves rapidly freezing the sample in a thin layer of vitrified ice and then imaging it using a transmission electron microscope [53]. CryoET allows studies of macromolecular structure and organization within cells or tissues [54]. Both techniques can provide unprecedented insights into the structure and function of biological systems, particularly for megadalton-scale biomolecular machines [55].

Following recommendations developed by the wwPDB 3DEM Validation Task Force [56] and community guidelines [28,57] for validation of structures generated using cryoEM and cryoET, we provide a set of numerical metrics and plots obtained from either EMDB [5] or calculated with the Validation Analysis (va) software [58] (Figure 2B). Assessment of the fit of a 3D structural model to 3DEM map data is currently available only for atomic structures.

The data quality criteria for 3DEM data include map visualization, map analysis, and Fourier-Shell Correlation (FSC) validation metrics (Supplementary Table 3A). Structural model criteria based on 3DEM data include map-model fit, Q-score, and atom inclusion (Supplementary Table 3B, Figure 2B).

## Local Geometry Assessment (i.e., Integrative Structure Model Quality)

### Stereochemistry

Analysis of structural model stereochemistry is well established for 3D biostructures coming from X-ray, NMR, or 3DEM [59]. This analysis highlights potential inaccuracies by identifying statistically significant deviations in stereochemical features, such as bond lengths and angles, from reference values derived from theory or from representative high-quality residues in high-resolution protein and nucleic acid structures (*e.g*., X-ray protein structures with resolution limits better than 2.0 Å) [60]. Care is needed, however, in interpreting outliers as either actual errors or accurate depictions that deviate from the norm [59]. While the stereochemistry of atomic integrative structure models can be assessed using existing tools, such as MolProbity [60], many integrative structure models are coarse-grained or multi-scale and cannot therefore be evaluated with existing tools.

We currently assess the stereochemistry of integrative structure models as follows. For components represented at atomic resolution, we follow the procedure adopted long ago by the wwPDB [61], relying on MolProbity [60]. In particular, we identify deviations of a model from accepted values for bond lengths, bond angles, dihedral angles, improper dihedral angles, and atomic overlaps. For coarse-grained and multi-scale components, we highlight overlaps between pairs of coarse-grained beads or between a coarse-grained bead and an atom. This test is analogous to estimating interatomic clashes within atomic models. A total excluded volume violation of a model is defined as the percentage of overlapping pairs of beads; a pair of beads is overlapping when the center-to-center distance between the beads is smaller than the sum of their radii, as specified by the depositor.

### Local model precision

If a structure is represented as a collection of conformations, we can define local model precision (uncertainty) as the corresponding local variability among the deposited models. This variability is quantified by the Precision for Integrative Structural Models (PrISM) program [62], which efficiently identifies and visualizes regions of high- and low-precision in an integrative structural model. While the depositor can provide already superposed models, superposition can also be performed by the validation pipeline. PrISM first computes root-mean-square fluctuations (RMSFs; spreads) of individual particles in the model collection, then partitions the model into volumes at several relative levels of precision using spatial clustering of particles with similar precisions. The validation report includes a visualization of high- and low-precision regions provided by PrISM (Figure 2D).

## Summary Table

To ensure open access to all information required to reproduce and evaluate integrative structure models, we have designed a validation summary table for integrative modeling, with input from the community. This table summarizes the input information, model representation, sampling protocol, software tools, and validation output in the human-readable PDF format (Supplementary File 2). The table is inspired by X-ray [29], NMR [30], EM [56], and SAS [24] validation summary tables. A validation summary table for every entry in the PDB-IHM is generated during the deposition. Depositors are encouraged to include these tables in their respective publications to supplement information available to editors, reviewers, and readers. A recently published integrative structure model (9A05) supplemented with such a table provides a proof-of-concept [63].

## Implementation

The IHMValidation pipeline is implemented as a Python package. To simplify dependency management for the third-party validation software (*e.g.*, MolProbity [60], ATSAS [64], mzidentml-reader, va [58], and PrISM [62]) and deployment, the IHMValidation pipeline is executed in an Apptainer (previously Singularity [65]) virtual container. The IHMValidation standalone validation server has been built using the open source DERIVA [66] scientific asset management platform.

## Future Directions

Future expansions of the IHMValidation pipeline will (i) reflect additional recommendations, including those from the Förster resonance energy transfer (FRET) and hydrogen/deuterium exchange MS (HDX MS) communities; (ii) include additional validation criteria, such as Bayesian likelihoods for the data given the structure; and (iii) represent estimates of structure uncertainty based on the variability among alternative models satisfying input information. We also welcome community contributions in the form of new methods and tools for the assessment of integrative structure models. We are committed to incorporating standards and tools for assessing integrative structure models and data as soon as corresponding communities develop them.

## Supplementary Material

Supplementary File 3

Supplementary File 2

Supplementary File 1

## Data Availability

Summary tables and validation reports are available in PDF and HTML formats on the PDB IHM website (https://pdb-ihm.org) and in the PDB archive (PDF only). The validation pipeline is available for standalone use at https://validate.pdb-ihm.org. The source code can be downloaded from the https://github.com/salilab/IHMValidation repository. The software has also been deployed within the PDB-IHM deposition system, enabling depositors to access validation reports after curation is complete.

## Appendix A. Supplementary material

Supplementary material to this article can be found online at https://doi.org/10.1016/j.jmb.2025.169598.
