# Supplementary material for: IHMValidation: Assessment of Integrative Structure Models Deposited to the Protein Data Bank
Source: J Mol Biol. Author manuscript; Available in PMC 2026 Apr 30. (PMC13126976; doi:10.1016/j.jmb.2025.169598)
Supplement: Supplementary File 3 [file NIHMS2167899-supplement-Supplementary_File_3.docx]

# Supplementary Data

## Tables

**Supplementary Table 1A.** List of SAS data validation criteria.

| **Criterion** | **Description** |
| --- | --- |
| Scattering profiles | Scattering data from solutions of biological macromolecules are presented as both log *I*(*q*) *vs* *q* and log *I*(*q*) *vs* log (*q*). The *I*(*q*) is the scattering intensity (preferably on an absolute scale in cm^-1^, but arbitrary units are accepted) and *q* is the modulus of the scattering vector (nm^-1^ or Å^-1^). |
| Experimental estimates | Molecular weight (MW) and volume data are displayed. Theoretical MW can be compared to SAS-derived values using the forward scatter (I(0)) and the known concentration and partial specific volume of the scattering particle, or as estimated from the Porod volume and partial specific volume [[1,2]](https://paperpile.com/c/2oypy1/UeCof+8w9qQ). |
| Porod-Debye plots | In a Porod-Debye plot, a clear plateau is observed for globular (partial or fully folded) domains, whereas flexible-modular, fully unfolded domains or extended/stiff rod-shaped domains lack a discernible plateau [[3]](https://paperpile.com/c/2oypy1/lnXpE). |
| Kratky plots | A bell-shaped Kratky plot (*q*^2^*I*(*q*) vs *q*) with a well-defined maximum is observed for compact/ folded structures. For partially flexible/modular or extended structures, the Kratky plot can show multiple maxima and/or an increase in intensity at higher *q*-values depending on the degree of flexibility and extension. Fully intrinsically disordered structures yield a Kratky plot that systematically increases with increasing *q values* and will be near linear for highly extended molecules. The dimensionless Kratky plot ((*qR_g_)*^2^*I*(*q*) vs *qR_g_*) is useful for quantifying differences in shape and foldedness among scattering objects of different sizes [[2]](https://paperpile.com/c/2oypy1/8w9qQ). |
| P(r) analysis | The PDDF or *P*(*r*) represents the distribution of distances between all pairs of atoms within the particle weighted by the respective scattering contrasts [[4]](https://paperpile.com/c/2oypy1/0CfjE). The second moment of *P*(*r*) yields the radius of gyration (*R_g_*), which is a measure of the overall size and shape of a macromolecule (*i.e.,* the spatial distribution of volume elements). A protein with a smaller *R_g_* is more compact than a protein with a larger *R_g_*, provided both have the same molecular weight. |
| Guinier analysis | The linearity of the Guinier plot (ln(*q*) vs. *q*^2^) at very-low angle (*qR_g_* < 1.3) is a sensitive indicator of the quality of the sample in relation to its homogeneity; a linear Guinier plot is a necessary but not sufficient demonstration that a solution contains monodisperse particles of the same size. Deviations from linearity can point to strong interference effects from particle attraction or repulsion, polydispersity of the samples, or improper background subtraction [[5]](https://paperpile.com/c/2oypy1/UU4jI). Residual difference plots and Pearson correlation coefficient determination (*R*^2^) are measures to assess the quality of the linear fit to the Guinier region. A perfect fit has an *R*^2^ value of 1. Residual values should be equally and randomly spaced around the horizontal axis with no evident systematic upward or downward curvature.  Agreement between the *P*(*r*) and Guinier-determined *R_g_* is a good measure of the self-consistency of the SAS profile. |

**Supplementary Table 1B.** List of the fit of the model to the SAS data criteria.

| **Criterion** | **Description** |
| --- | --- |
| Model versus Experimental Scattering Profiles | Experimental and model scattering profiles are presented as log *I*(*q*) *vs* *q* together with error-weighted residual difference plot between the experimental and model *I(q) vs q*. The *I*(*q*) is the intensity (preferably in absolute units of cm^-1^ or arbitrary units) and *q* is the modulus of the scattering vector. For a good fit, residual values should be equally and randomly spaced around the horizontal axis, largely within +/- three standard deviations [[1]](https://paperpile.com/c/2oypy1/UeCof). |
| χ² | *χ²* values are a measure of the overall fit of the model to the 1D scattering profile. A model that fits the data within its error estimates will have a *χ²* value close to one, provided that the dominant errors are the random statistical errors (*i.e.,* no systematic errors) from the SAS measurement that are correctly propagated [[6–8]](https://paperpile.com/c/2oypy1/C3bux+y64DM+zXGhW). |
| CorMap Test | Correlation Map (CorMap) test [[9]](https://paperpile.com/c/2oypy1/bgakR) is a variance-covariance analysis on the scattering intensities comparing two (or more) scattering profiles (*e.g.,* model versus experiment or multiple measures from the same sample). The CorMap test complements *χ²* and, importantly, is independent of the reported errors. The method assigns a probability (*P*-value based on a 1-tailed Schilling test) for finding the longest string of experimental data points that lie systematically above (+1) or below (-1) the model profile. The *P*-value lies between 0 and 1, and a significance threshold is chosen below which the model fit is judged to show systematic deviation from experiment. A typical range statisticians use to indicate significant deviation is 0.01 - 0.05. As implemented in the ATSAS [[10]](https://paperpile.com/c/2oypy1/nxG4) suite, the reported CorMap *P*-value is green (model fit is good) for *P* > 0.05, yellow for 0.01<P<0.05, and red (model deviates significantly) for P< 0.01. |

**Supplementary Table 2A.** List of crosslinking-MS data validation criteria.

| **Criterion** | **Description** |
| --- | --- |
| Data completeness | It shows how many experimentally-detected crosslinks for given entities were actually used for modeling. We compare entities in the MS search database with those reported in the mmCIF file and match corresponding crosslinks. The values are reported as percentages of crosslinks present in the data and have to be interpreted in the context of the experiment (*i.e.,* only a minor fraction of an *in situ* or *in vivo* dataset can be used for modeling). |

**Supplementary Table 2B.** List of the fit of the model to the crosslinking-MS data criteria.

| **Criterion** | **Description** |
| --- | --- |
| Restraint types | This table summarizes information about crosslinker(s) used for data generation, and how crosslinking information was translated into actual modeling restraints. |
| Distogram plots | Distograms (*i.e.*, histogram plots of distances) provide an overview of distributions of distances between residues for which chemical crosslinks were identified. The shift of the distogram relative to the threshold value may indicate a poor model. |
| Satisfaction rates | Numerical crosslink satisfaction rates are calculated based on the threshold values and restraint types. Rates are calculated per model (or collection of models, where applicable) and per entity type. |

**Supplementary Table 3A.** List of 3DEM data validation criteria.

| **Criterion** | **Description** |
| --- | --- |
| **Map visualization** | |
| Orthogonal projections | The images show the map projected in three orthogonal directions, in greyscale. |
| Central slices | The images show the central slice of the map in three orthogonal projections. |
| Largest variance slices | The images show the largest variance slices of the map in three orthogonal directions. The index of the slice in the relevant axis is given below the image. |
| Orthogonal standard-deviation projections | The images show the map projected in three orthogonal directions in false color. |
| Orthogonal surface views | The images show the 3D surface of the map at the recommended contour level. These images, in conjunction with the slice images, may facilitate assessment of whether an appropriate contour level has been provided. |
| Mask visualization | This section shows the 3D surface view of the primary map overlaid with the specified mask. A mask typically either encompasses the whole structure and indicates the removal of noise from the periphery of the map, or separates out a domain, a functional unit, a monomer, or an area of interest from the larger structure. |
| **Map analysis** | |
| Map value distribution | The distribution of voxel values in the map. A spike at around 0 usually indicates that the volume has been masked. |
| Volume estimate by contour | The volume estimate graph shows how the enclosed volume varies with contour level. |
| Rotationally averaged power spectrum | The rotationally averaged power spectrum (RAPS) may provide insight into the data processing steps leading to the map, in terms of (i) CTF correction, (ii) Temperature factor correction, (iii) Low and/or high-pass filtering, and (iv) Masking artifacts. |
| **Fourier-Shell Correlation (FSC) validation** | |
| FSC | Graph of the FSC curve(s). |
| Resolution estimate | Resolution estimates for the map based on different criteria. |

**Supplementary Table 3B.** List of the fit of the model to the 3DEM data criteria.

| **Criterion** | **Description** |
| --- | --- |
| Map-model fit figures | The images show the 3D surface view of the map at the recommended contour level with a ribbon representation of the model. |
| Q-score | The images show the Q-score for each residue mapped to the coordinate model. |
| Atom inclusion | The images show atom inclusion for each residue mapped to the coordinate mode. |

#

# References

[1] [J. Trewhella, A.P. Duff, D. Durand, F. Gabel, J.M. Guss, W.A. Hendrickson, G.L. Hura, D.A. Jacques, N.M. Kirby, A.H. Kwan, J. Pérez, L. Pollack, T.M. Ryan, A. Sali, D. Schneidman-Duhovny, T. Schwede, D.I. Svergun, M. Sugiyama, J.A. Tainer, P. Vachette, J. Westbrook, A.E. Whitten, 2017 publication guidelines for structural modelling of small-angle scattering data from biomolecules in solution: an update, Acta Crystallogr D Struct Biol 73 (2017) 710–728. https://doi.org/](http://paperpile.com/b/2oypy1/UeCof)[10.1107/S2059798317011597.](http://dx.doi.org/10.1107/S2059798317011597.)

[2] [J. Trewhella, C.M. Jeffries, A.E. Whitten, 2023 update of template tables for reporting biomolecular structural modelling of small-angle scattering data, Acta Crystallogr. D Struct. Biol. 79 (2023) 122–132. https://doi.org/](http://paperpile.com/b/2oypy1/8w9qQ)[10.1107/S2059798322012141.](http://dx.doi.org/10.1107/S2059798322012141.)

[3] [R.P. Rambo, J.A. Tainer, Characterizing flexible and intrinsically unstructured biological macromolecules by SAS using the Porod-Debye law, Biopolymers 95 (2011) 559–571. https://doi.org/](http://paperpile.com/b/2oypy1/lnXpE)[10.1002/bip.21638.](http://dx.doi.org/10.1002/bip.21638.)

[4] [P.B. Moore, Small-angle scattering. Information content and error analysis, J. Appl. Crystallogr. 13 (1980) 168–175. https://doi.org/](http://paperpile.com/b/2oypy1/0CfjE)[10.1107/s002188988001179x.](http://dx.doi.org/10.1107/s002188988001179x.)

[5] [L.A. Feigin, D.I. Svergun, Structure analysis by small-angle X-ray and neutron scattering, Springer, New York, NY, 2013. https://doi.org/](http://paperpile.com/b/2oypy1/UU4jI)[10.1007/978-1-4757-6624-0.](http://dx.doi.org/10.1007/978-1-4757-6624-0.)

[6] [J. Trewhella, W.A. Hendrickson, G.J. Kleywegt, A. Sali, M. Sato, T. Schwede, D.I. Svergun, J.A. Tainer, J. Westbrook, H.M. Berman, Report of the wwPDB Small-Angle Scattering Task Force: data requirements for biomolecular modeling and the PDB, Structure 21 (2013) 875–881. https://doi.org/](http://paperpile.com/b/2oypy1/C3bux)[10.1016/j.str.2013.04.020.](http://dx.doi.org/10.1016/j.str.2013.04.020.)

[7] [D. Schneidman-Duhovny, S.J. Kim, A. Sali, Integrative structural modeling with small angle X-ray scattering profiles, BMC Struct. Biol. 12 (2012) 17. https://doi.org/](http://paperpile.com/b/2oypy1/y64DM)[10.1186/1472-6807-12-17.](http://dx.doi.org/10.1186/1472-6807-12-17.)

[8] [R.P. Rambo, J.A. Tainer, Accurate assessment of mass, models and resolution by small-angle scattering, Nature 496 (2013) 477–481. https://doi.org/](http://paperpile.com/b/2oypy1/zXGhW)[10.1038/nature12070.](http://dx.doi.org/10.1038/nature12070.)

[9] [D. Franke, C.M. Jeffries, D.I. Svergun, Correlation Map, a goodness-of-fit test for one-dimensional X-ray scattering spectra, Nat. Methods 12 (2015) 419–422. https://doi.org/](http://paperpile.com/b/2oypy1/bgakR)[10.1038/nmeth.3358.](http://dx.doi.org/10.1038/nmeth.3358.)

[10] [K. Manalastas-Cantos, P.V. Konarev, N.R. Hajizadeh, A.G. Kikhney, M.V. Petoukhov, D.S. Molodenskiy, A. Panjkovich, H.D.T. Mertens, A. Gruzinov, C. Borges, C.M. Jeffries, D.I. Svergun, D. Franke, : expanded functionality and new tools for small-angle scattering data analysis, J Appl Crystallogr 54 (2021) 343–355. https://doi.org/](http://paperpile.com/b/2oypy1/nxG4)[10.1107/S1600576720013412.](http://dx.doi.org/10.1107/S1600576720013412.)
