# Supplementary material for: IHMValidation: Assessment of Integrative Structure Models Deposited to the Protein Data Bank
Source: J Mol Biol. Author manuscript; Available in PMC 2026 Apr 30. (PMC13126976; doi:10.1016/j.jmb.2025.169598)
Supplement: Supplementary File 2 [file NIHMS2167899-supplement-Supplementary_File_2.pdf]

# Summary of integrative structure determination of Modeling hLINE1 ORF2p (PDB ID: 9A3Q | pdb_00009a3q, PDB-Dev ID: PDBDEV_00000211 )

| *1. Model Composition* | |
|---|---|
| *1.1. Entry composition* | ORF2: chain(s) A (1275 residues) |
| *1.2. Datasets used for modeling* | - De Novo model, AlphaFoldDB: AF-O00370-F1<br>- De Novo model, MODEL ARCHIVE: ma-fejd6<br>- De Novo model, MODEL ARCHIVE: ma-joo4d<br>- De Novo model, MODEL ARCHIVE: ma-lzyrq<br>- De Novo model, MODEL ARCHIVE: ma-xlzzy<br>- Mass Spectrometry data, PRIDE: PXD038615<br>- Crosslinking-MS data, Zenodo: 10.5281/zenodo.10377421<br>- Crosslinking-MS data, Zenodo: 10.5281/zenodo.10377421<br>- Crosslinking-MS data, Zenodo: 10.5281/zenodo.10377421<br>- EM raw micrographs, EMPIAR: EMPIAR-11556<br>- 3DEM volume, EMDB: 40856<br>- 3DEM volume, Zenodo: 10.5281/zenodo.10377421<br>- De Novo model, MODEL ARCHIVE: ma-9wovj<br>- 2DEM class average, Zenodo: 10.5281/zenodo.10377421<br>- 2DEM class average, Zenodo: 10.5281/zenodo.10377421<br>- 2DEM class average, Zenodo: 10.5281/zenodo.10377421 |
| ***2. Representation*** | |
| *2.1. Number of representations* | 1 |
| *2.2. Scale* | Coarse-grained: 1 residue(s) per bead |
| *2.3. Number of rigid and flexible segments* | 15, 14 |
| ***3. Restraints*** | |
| *3.1. Physical principles* | Information about physical principles was not provided |
| *3.2. Experimental data* | - 1 unique EM2DRestraint: Number of micrographs: None, Image resolution: 20.0<br>- 1 unique EM3DRestraint: Gaussian mixture models<br>- 1 unique CrossLinkRestraint: BS3, 15 crosslinks<br>- 1 unique CrossLinkRestraint: BS3, 11 crosslinks<br>- 1 unique CrossLinkRestraint: BS3, 30 crosslinks<br>- 15 unique PredictedContactRestraint: Distance: 27.0 |
| ***4. Validation*** | |
| *4.2. Number of ensembles* | 1 |
| *4.3. Number of models in ensembles* | 1383 |

| | |
|---|---|
| *4.4. Number of deposited models* | 159 |
| *4.5. Model precision* | 12.87, Å |
| *4.6. Data quality* | 40856: resolution is 4.06 Å |
| *4.7. Model quality: assessment of excluded volume* | Satisfaction: 99.60-99.61% |
| *4.8. Fit to data used for modeling* | Satisfaction of crosslinks: 85.71-92.86% |
| *4.9. Fit to data used for validation* | Fit of model to information not used to compute it has not been determined |
| ***5. Methodology and Software*** | |
| *1. 5.1. Method name* | Sampling |
| *5.2. Method type* | AlphaFold2 |
| *5.3. Method description* | Modeling of full-length ORF2p with AlphaFold2 using varying alignment depth. Details of the simulations are availabel in the ModelArchive entry ma-fejd6 |
| *2. 5.1. Method name* | Sampling |
| *5.2. Method type* | Molecular Dynamics simulations |
| *5.3. Method description* | Details of molecular dynamics simulations are available ModelArchive entries |
| *3. 5.1. Method name* | Sampling |
| *5.2. Method type* | Replica Exchange Gibbs sampling, based on Metropolis Monte Carlo |
| *5.3. Method description* | 20 replicas; 3 runs; 10000 models per run |
| *5.4. Number of computed models* | 30000 |
| *4. 5.1. Method name* | Refinement |
| *5.2. Method type* | Steepest descent |
| *5.3. Method description* | Conversion of a Ca-model to a full backbone model |
| *5.4. Number of computed models* | 159 |
| *5. 5.1. Method name* | Refinement |
| *5.2. Method type* | SCWRL |
| *5.3. Method description* | Conversion of a backbone model to afull-atom model |
| *5.4. Number of computed models* | 159 |

| | |
|---|---|
| *6. 5.1. Method name* | Refinement |
| *5.2. Method type* | Geometry optimization |
| *5.3. Method description* | Conversion of a backbone model to a full-atom model |
| *5.4. Number of computed models* | 159 |
| *5.5. Software* | - Sampcon (version 2.18.0)<br>- ColabFold (version 1.3.0)<br>- GROMACS (version 2022.3)<br>- IMP PMI module (version 2.19.0)<br>- PULCHRA (version 3.04)<br>- SCWRL4.0 (version 4.0)<br>- Integrative Modeling Platform (IMP) (version 2.19.0) |
