# Supplementary material for: IHMValidation: Assessment of Integrative Structure Models Deposited to the Protein Data Bank
Source: J Mol Biol. Author manuscript; Available in PMC 2026 Apr 30. (PMC13126976; doi:10.1016/j.jmb.2025.169598)
Supplement: Supplementary File 1 [file NIHMS2167899-supplement-Supplementary_File_1.pdf]

Integrative Structure Validation Report ?

October 09, 2025 - 04:43 PM PDT

The following software was used in the production of this report:

IHMValidation Version 3.0
Python-IHM Version 2.5
EMDB validation analysis Version 0.0.1.dev127
ChimeraX Version 1.9
Chimera Version 1.19
MapQ Version 1.8.1
PrISM Version dbe5a41
PyMOL Version 2.5.0

PDB ID	9A3Q | pdb_00009a3q
PDB-Dev ID	PDBDEV_00000211
Structure Title	Modeling hLINE1 ORF2p
Structure Authors	Baldwin, E.T.; van Eeuwen, T.; Hoyos, T.; Zalevsky, A.; Tchesnokov, E.P; Sanchez, R.; DiStefano, L.; Ruiz, F.X; Hancock, M.; Walpole, T.; Nichols, C.; Wan, P.; Riento, K.; Kass, R.-H.; Augustin, M.; Lammens, A.; Jestel, A.; Upla, P.; Xibinaku, K.; Congreve, S.; Hennink, M,; Rogala, K.B.; Schneider, A.M.; Fairman, J.E.; Christensen, S.M.; Miao, W.; Zaller, D.M.; Sali, A.; Weichenrieder, O.; Burns, K.H.; Gotte, M.; Rout, M.P.; Arnold, E.; Greenbaum, B.D.; Romero, D.L.; LaCava, J.; Taylor, M.S.
Deposited on	2023-05-25

This is a PDB-IHM Structure Validation Report.

We welcome your comments at helpdesk@pdb-ihm.org

A user guide is available at https://pdb-ihm.org/validation_help.html with specific help available everywhere you see the ? symbol.

List of references used to build this report is available here.

1. Overview ?

1.1. Summary ?

This entry consists of 159 model(s). A total of 16 dataset(s) were used to build this entry.

Name	Type	Count
2DEM class average	Experimental data	3
3DEM volume	Experimental data	2
Crosslinking-MS data	Experimental data	3
EM raw micrographs	Experimental data	1
Mass Spectrometry data	Experimental data	1
De Novo model	Starting model	6

1.2. Overall quality

This validation report contains model quality assessments for all structures, data quality and fit to model assessments for SAS and crosslinking-MS datasets. Data quality and fit to model assessments for other datasets and model uncertainty are under development. Number of plots is limited to 256.

Model Quality: Excluded Volume Analysis

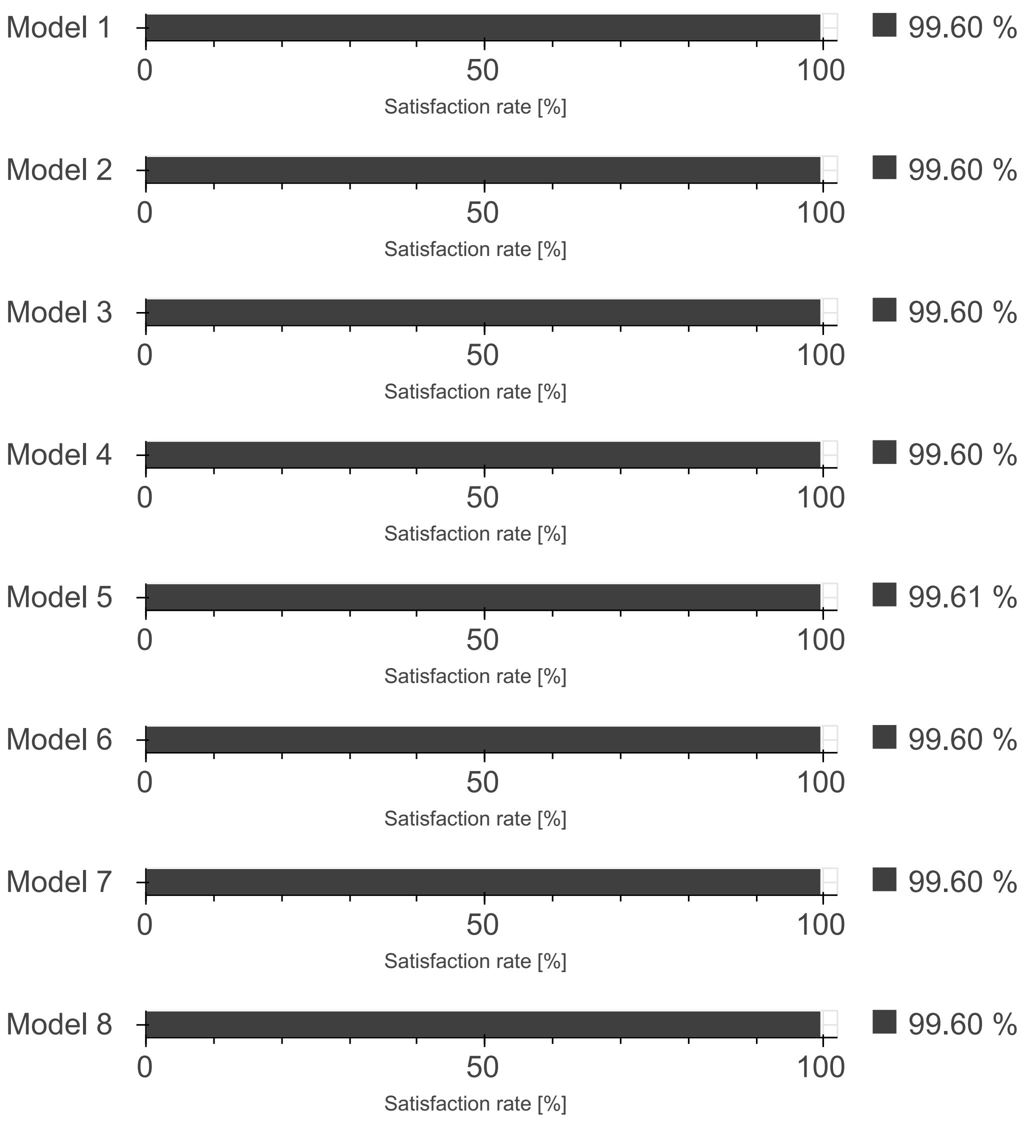

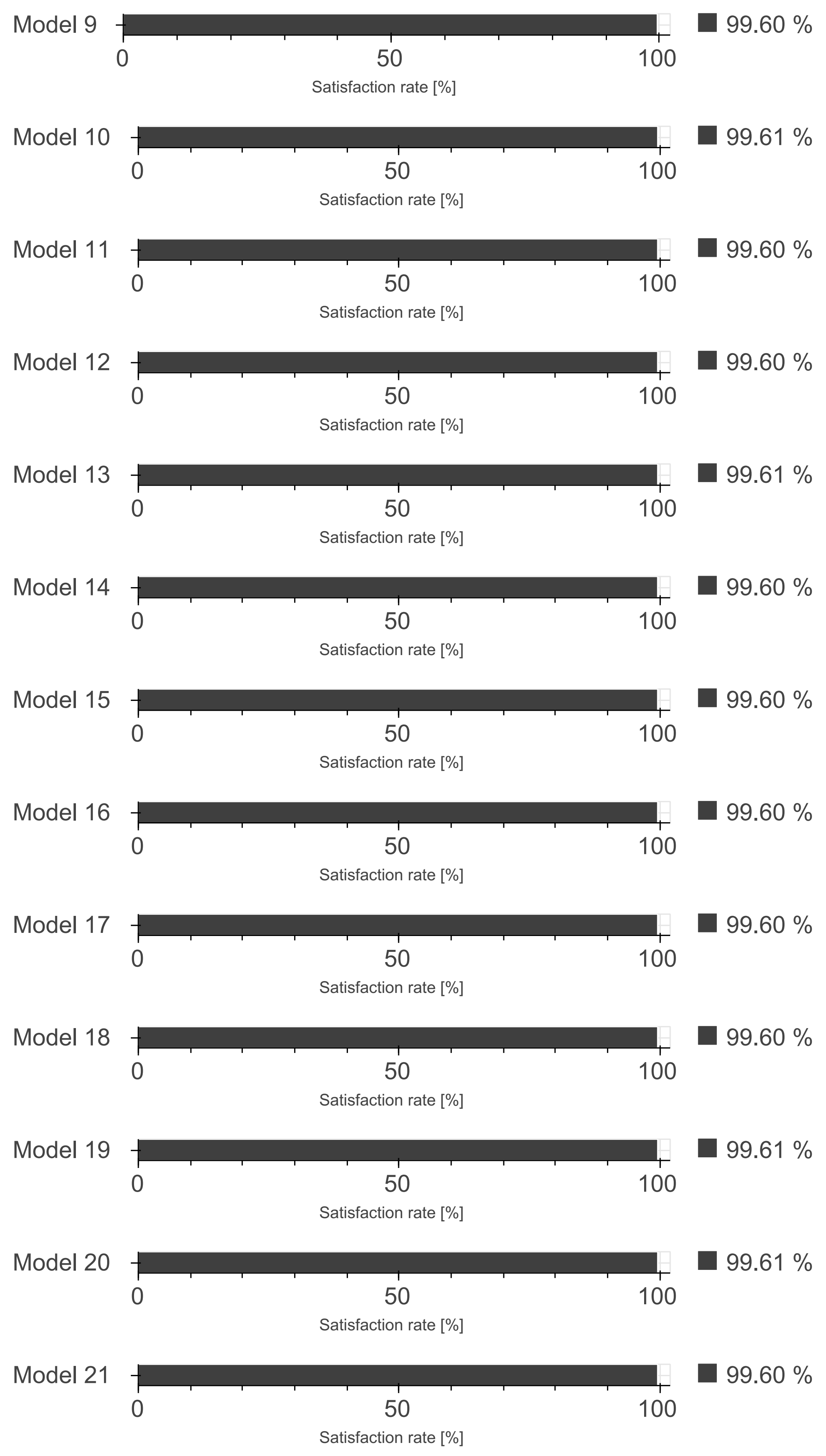
Model 9
99.60 %
Model 10
99.61 %
Model 11
99.60 %
Model 12
99.60 %
Model 13
99.61 %
Model 14
99.60 %
Model 15
99.60 %
Model 16
99.60 %
Model 17
99.60 %
Model 18
99.60 %
Model 19
99.61 %
Model 20
99.61 %
Model 21
99.60 %
0
50
100
Satisfaction rate [%]

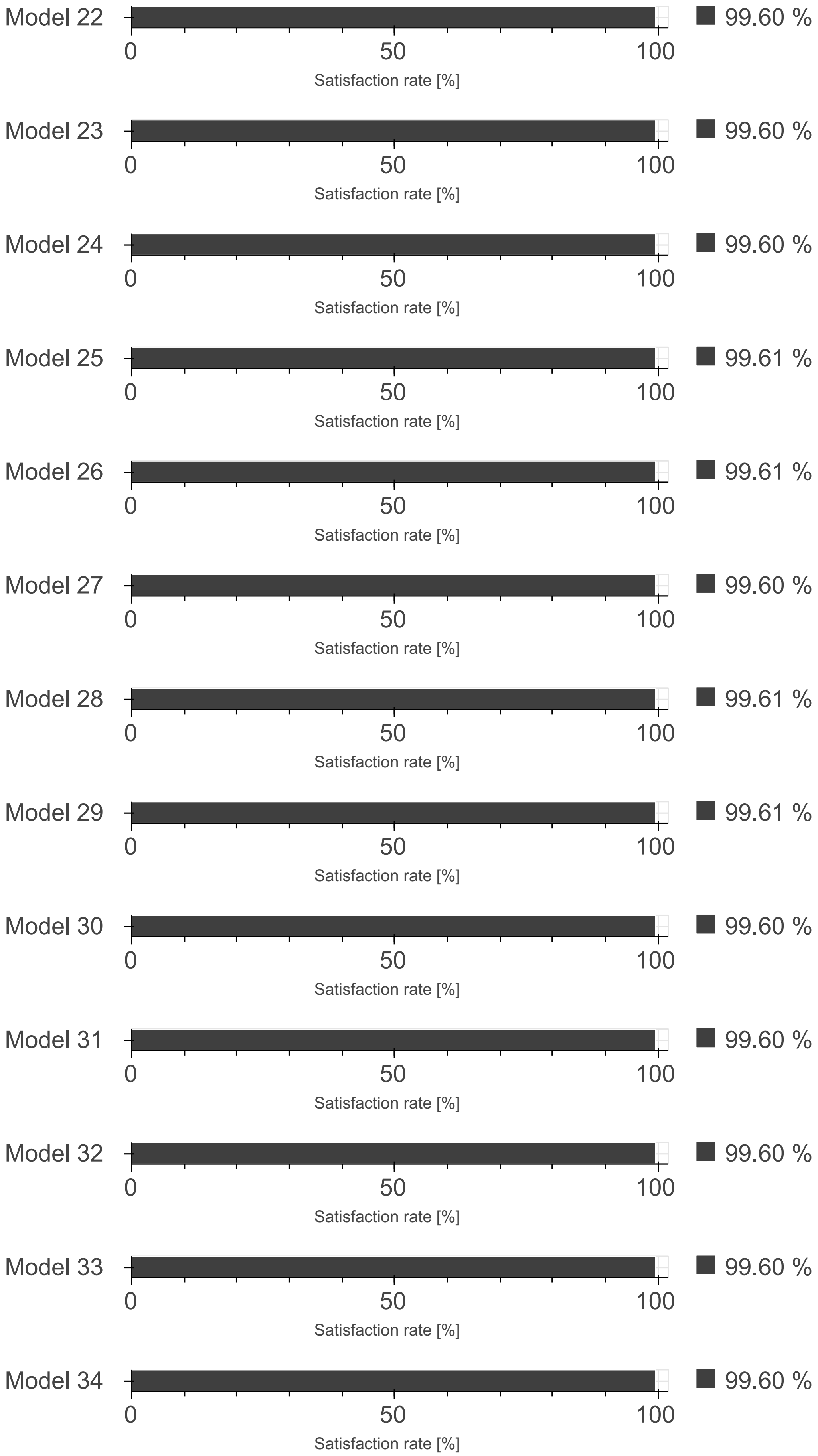
Model 22
99.60 %
Model 23
99.60 %
Model 24
99.60 %
Model 25
99.61 %
Model 26
99.61 %
Model 27
99.60 %
Model 28
99.61 %
Model 29
99.61 %
Model 30
99.60 %
Model 31
99.60 %
Model 32
99.60 %
Model 33
99.60 %
Model 34
99.60 %
0
50
100
Satisfaction rate [%]

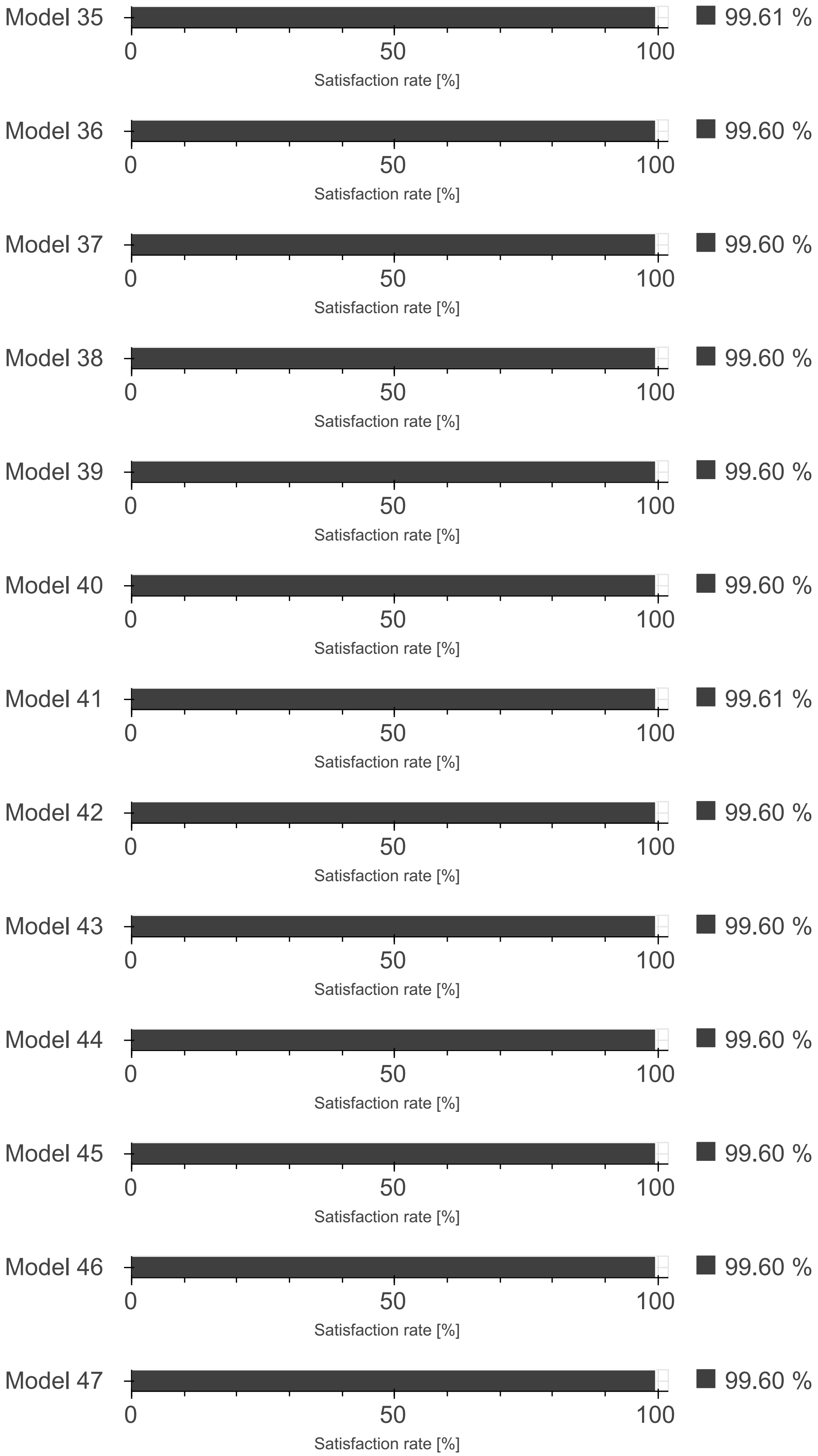
Model 35
99.61 %
Model 36
99.60 %
Model 37
99.60 %
Model 38
99.60 %
Model 39
99.60 %
Model 40
99.60 %
Model 41
99.61 %
Model 42
99.60 %
Model 43
99.60 %
Model 44
99.60 %
Model 45
99.60 %
Model 46
99.60 %
Model 47
99.60 %
0
50
100
Satisfaction rate [%]

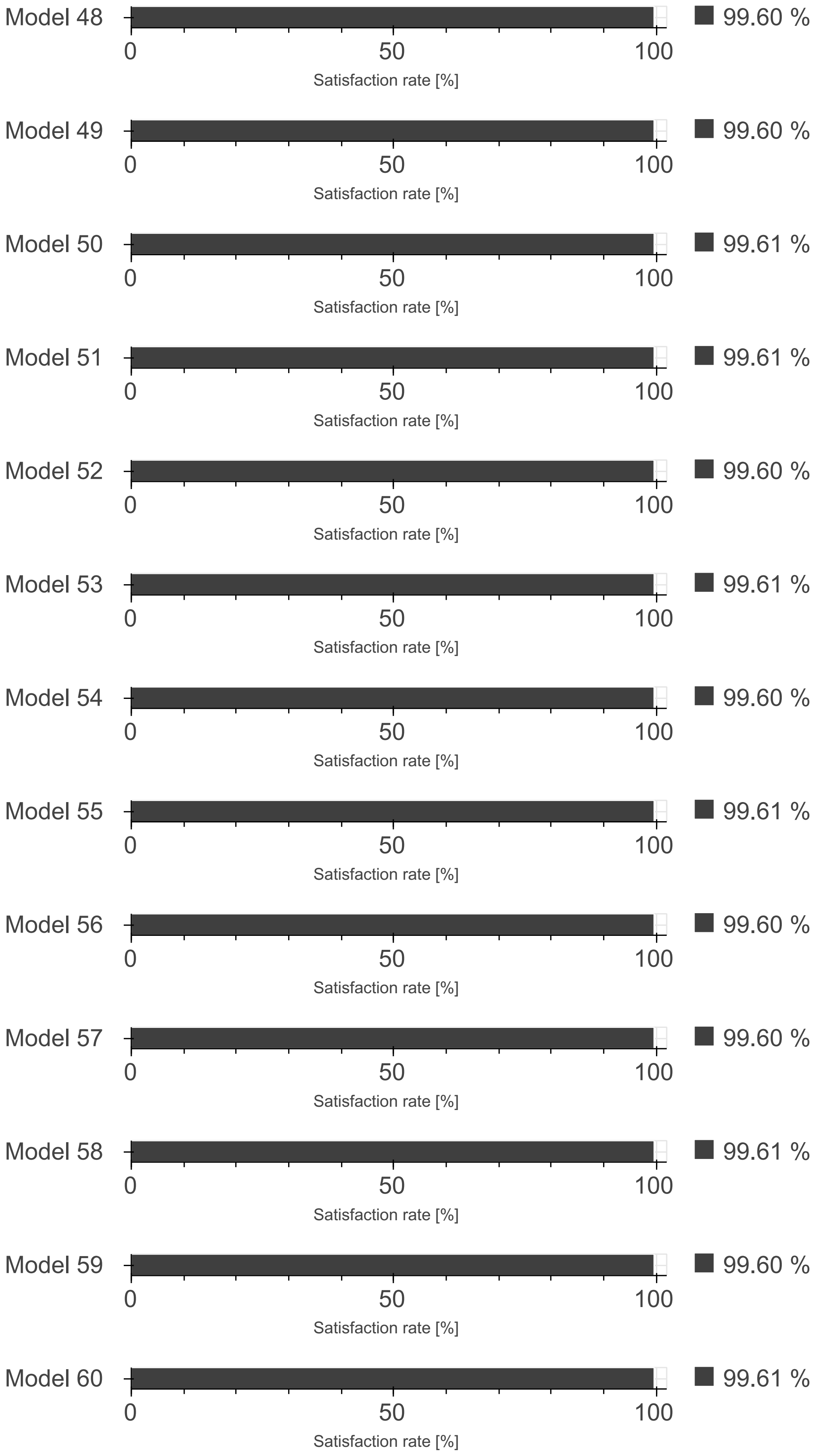
Model 48
99.60 %
Model 49
99.60 %
Model 50
99.61 %
Model 51
99.61 %
Model 52
99.60 %
Model 53
99.61 %
Model 54
99.60 %
Model 55
99.61 %
Model 56
99.60 %
Model 57
99.60 %
Model 58
99.61 %
Model 59
99.60 %
Model 60
99.61 %
0
50
100
Satisfaction rate [%]

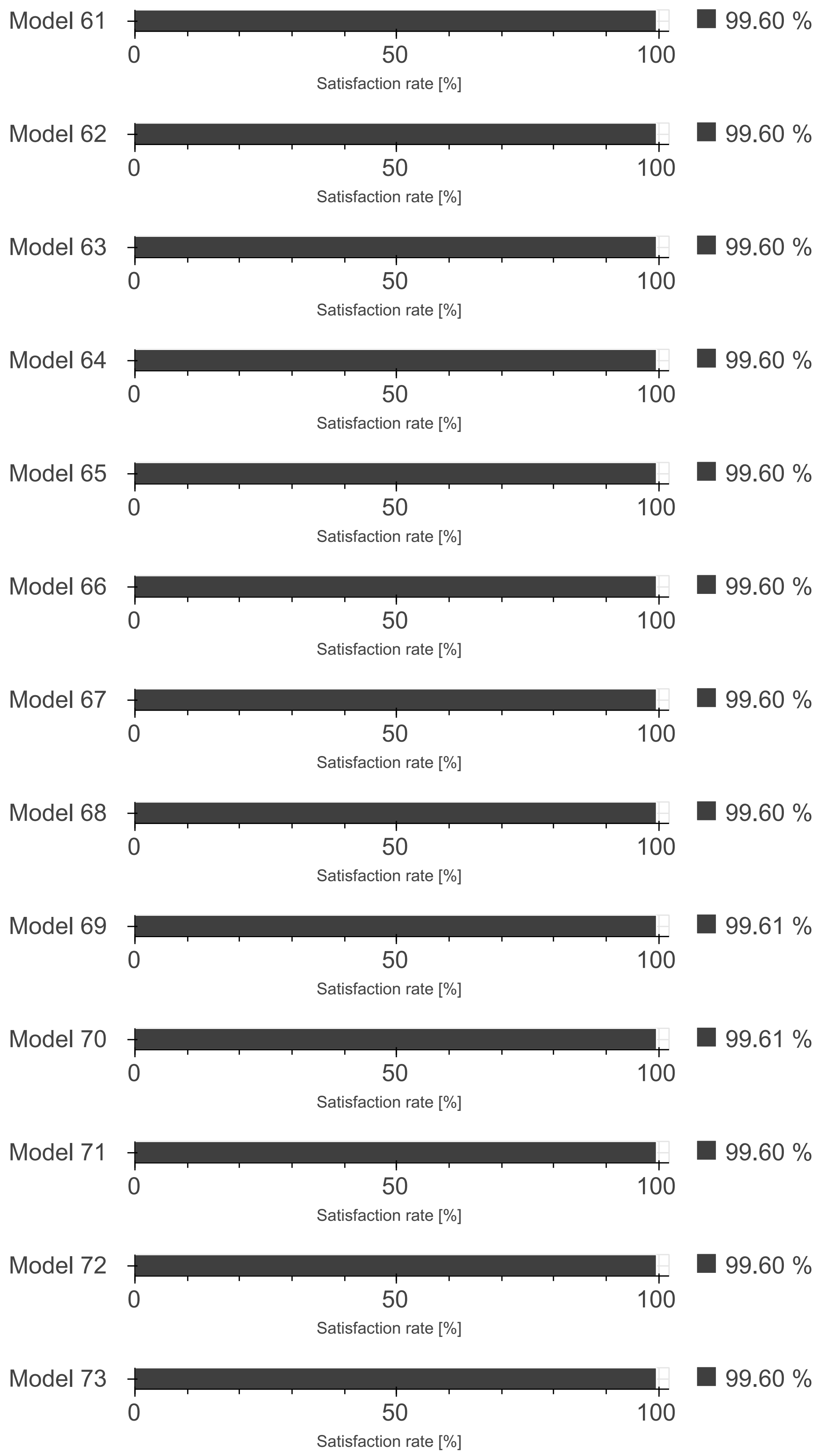
Model 61
99.60 %
Model 62
99.60 %
Model 63
99.60 %
Model 64
99.60 %
Model 65
99.60 %
Model 66
99.60 %
Model 67
99.60 %
Model 68
99.60 %
Model 69
99.61 %
Model 70
99.61 %
Model 71
99.60 %
Model 72
99.60 %
Model 73
99.60 %
0
50
100
Satisfaction rate [%]

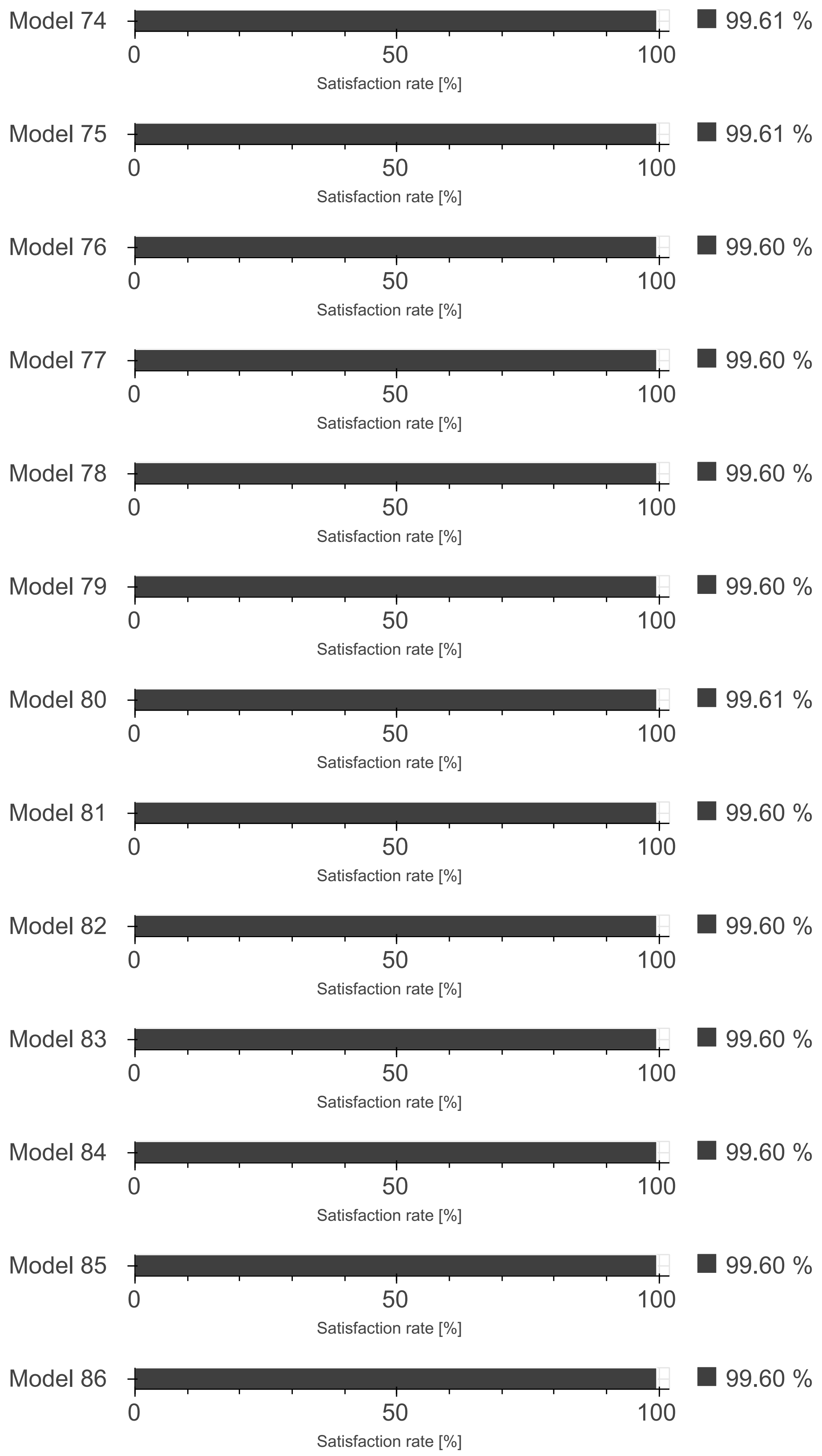
Model 74
0
50
100
Satisfaction rate [%]
99.61 %
Model 75
0
50
100
Satisfaction rate [%]
99.61 %
Model 76
0
50
100
Satisfaction rate [%]
99.60 %
Model 77
0
50
100
Satisfaction rate [%]
99.60 %
Model 78
0
50
100
Satisfaction rate [%]
99.60 %
Model 79
0
50
100
Satisfaction rate [%]
99.60 %
Model 80
0
50
100
Satisfaction rate [%]
99.61 %
Model 81
0
50
100
Satisfaction rate [%]
99.60 %
Model 82
0
50
100
Satisfaction rate [%]
99.60 %
Model 83
0
50
100
Satisfaction rate [%]
99.60 %
Model 84
0
50
100
Satisfaction rate [%]
99.60 %
Model 85
0
50
100
Satisfaction rate [%]
99.60 %
Model 86
0
50
100
Satisfaction rate [%]
99.60 %

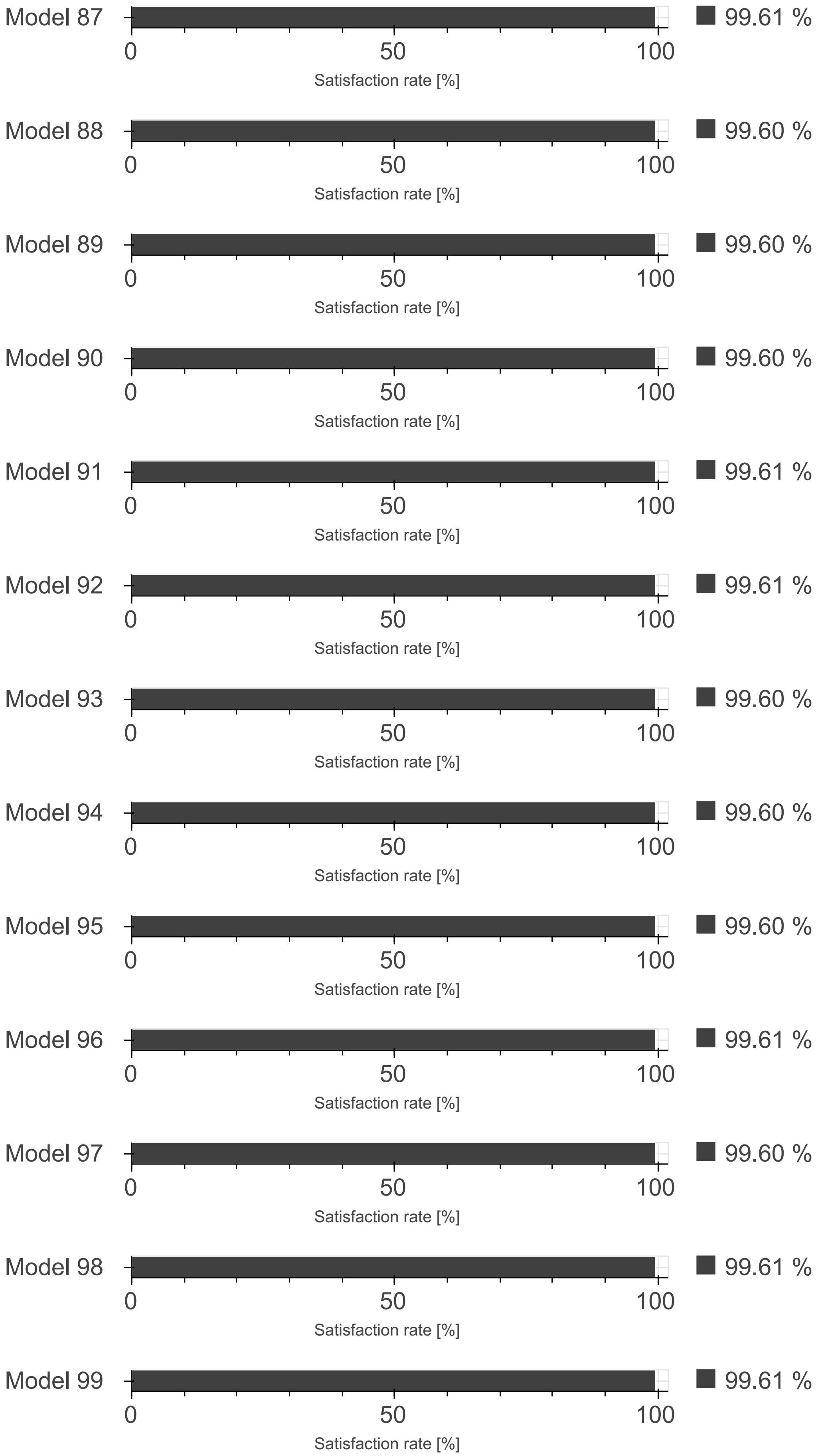
Model 87
99.61 %
Model 88
99.60 %
Model 89
99.60 %
Model 90
99.60 %
Model 91
99.61 %
Model 92
99.61 %
Model 93
99.60 %
Model 94
99.60 %
Model 95
99.60 %
Model 96
99.61 %
Model 97
99.60 %
Model 98
99.61 %
Model 99
99.61 %
0
50
100
Satisfaction rate [%]

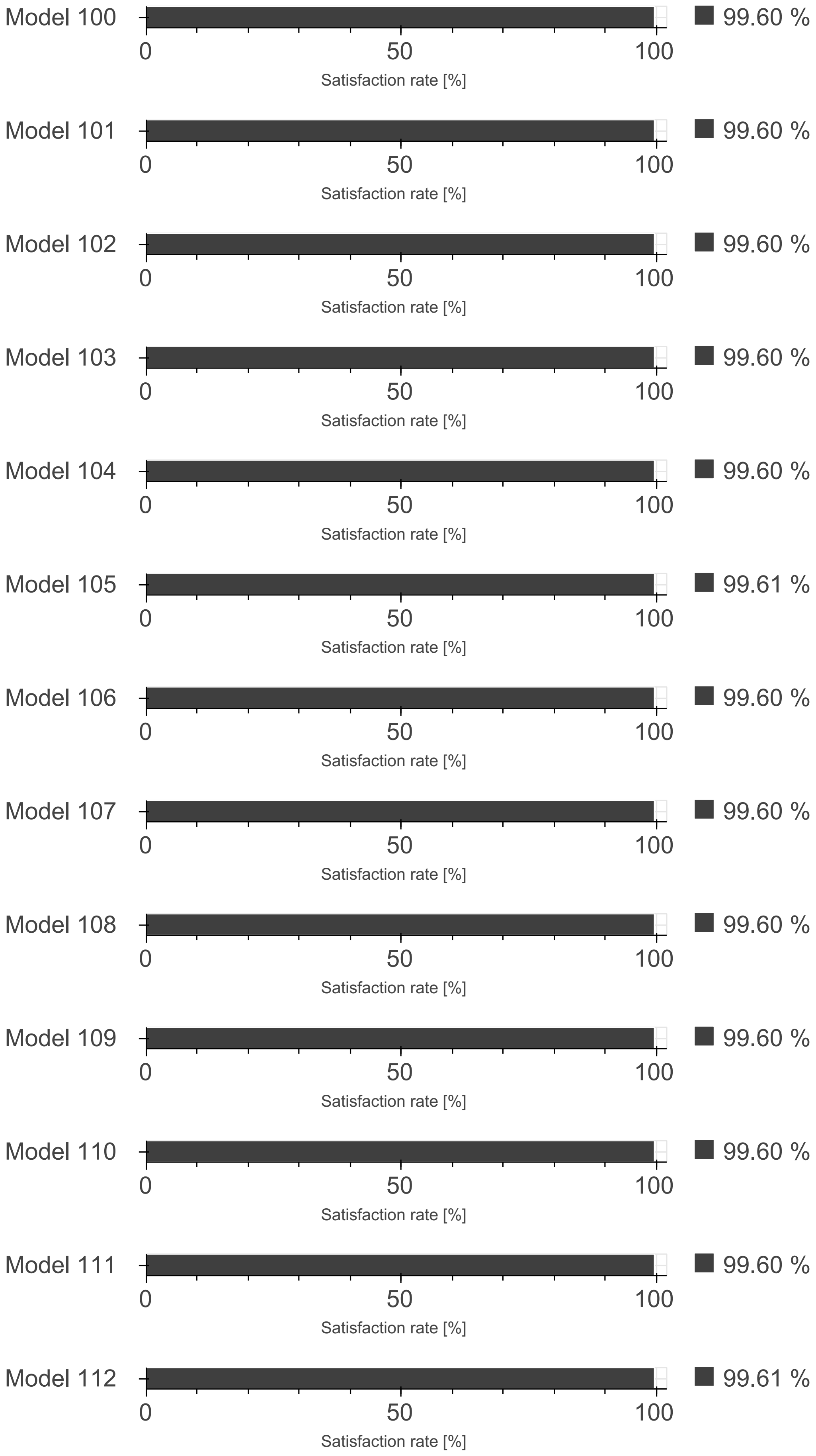
Model 100
99.60 %
Model 101
99.60 %
Model 102
99.60 %
Model 103
99.60 %
Model 104
99.60 %
Model 105
99.61 %
Model 106
99.60 %
Model 107
99.60 %
Model 108
99.60 %
Model 109
99.60 %
Model 110
99.60 %
Model 111
99.60 %
Model 112
99.61 %
0
50
100
Satisfaction rate [%]

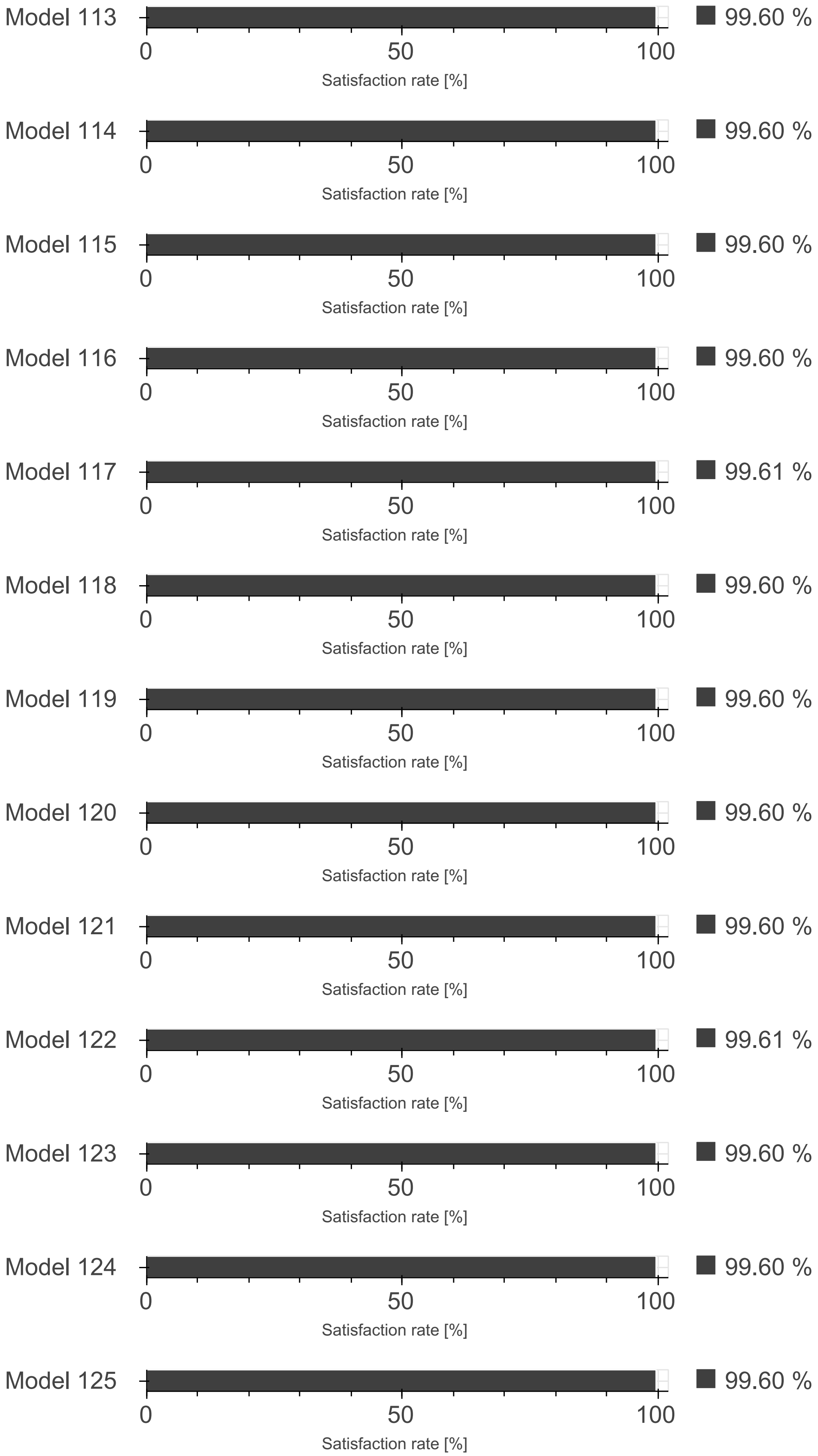
Model 113
99.60 %
Model 114
99.60 %
Model 115
99.60 %
Model 116
99.60 %
Model 117
99.61 %
Model 118
99.60 %
Model 119
99.60 %
Model 120
99.60 %
Model 121
99.60 %
Model 122
99.61 %
Model 123
99.60 %
Model 124
99.60 %
Model 125
99.60 %
0
50
100
Satisfaction rate [%]

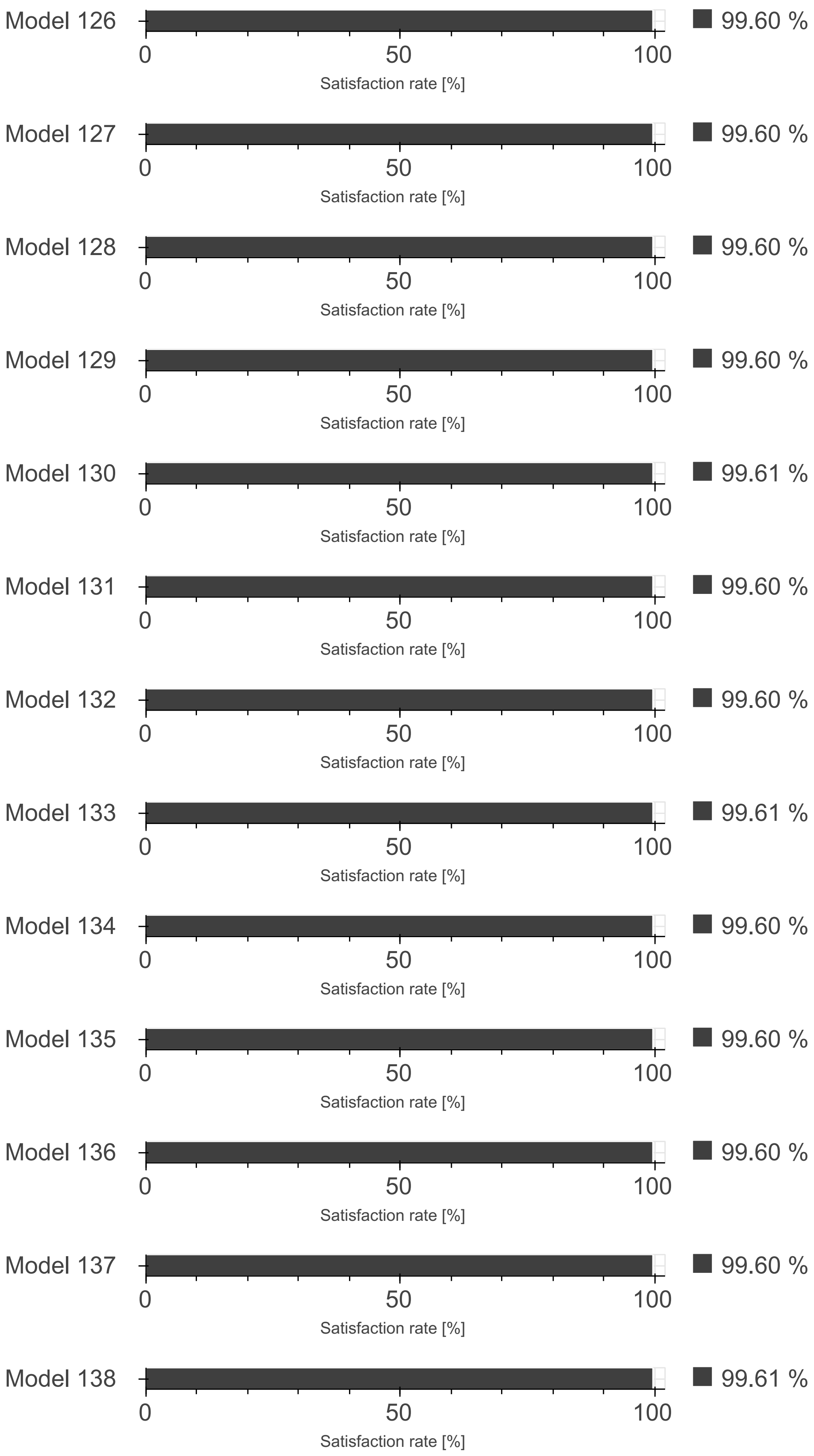
Model 126
99.60 %
Model 127
99.60 %
Model 128
99.60 %
Model 129
99.60 %
Model 130
99.61 %
Model 131
99.60 %
Model 132
99.60 %
Model 133
99.61 %
Model 134
99.60 %
Model 135
99.60 %
Model 136
99.60 %
Model 137
99.60 %
Model 138
99.61 %
0
50
100
Satisfaction rate [%]

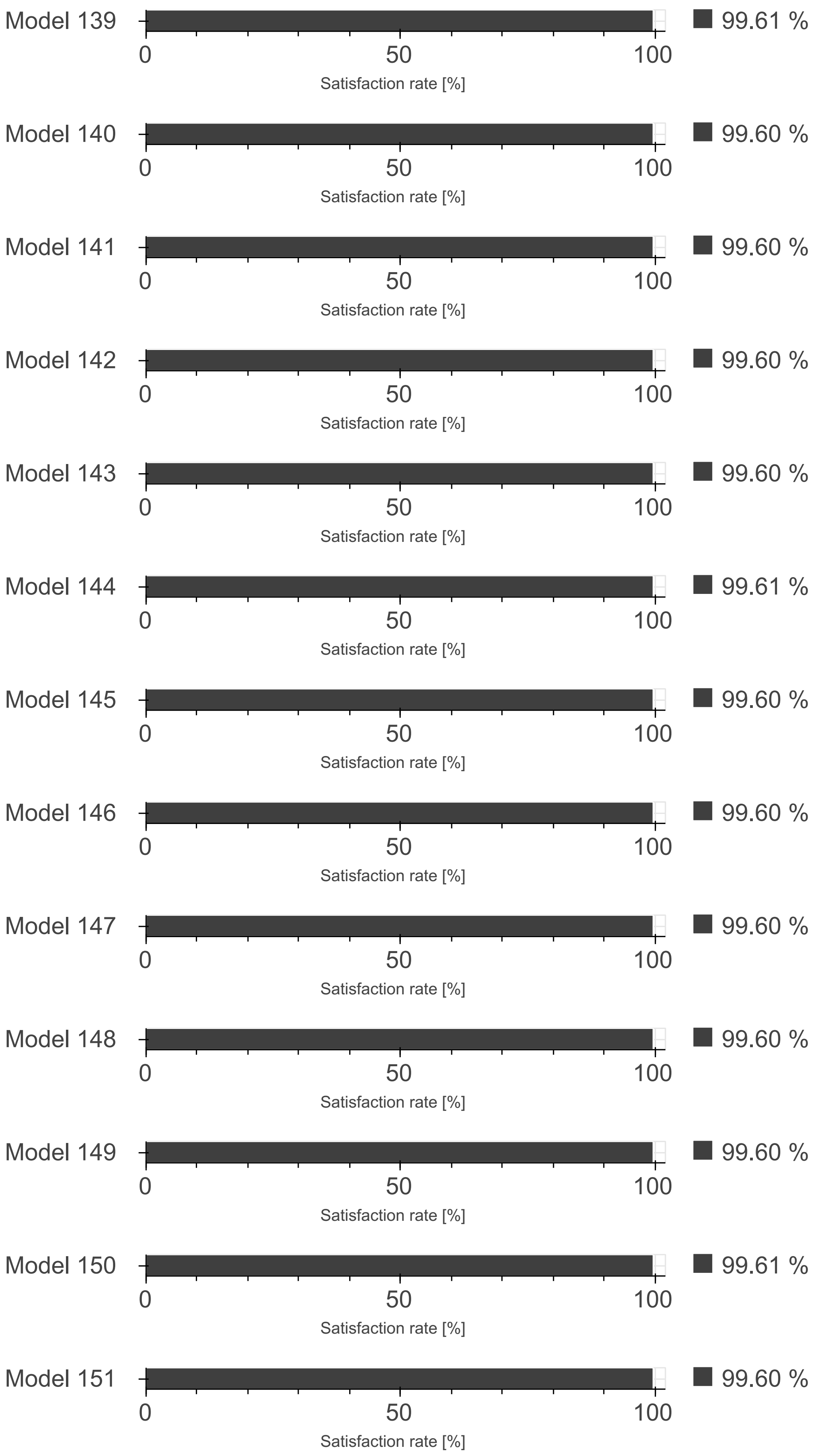
Model 139
99.61 %
Model 140
99.60 %
Model 141
99.60 %
Model 142
99.60 %
Model 143
99.60 %
Model 144
99.61 %
Model 145
99.60 %
Model 146
99.60 %
Model 147
99.60 %
Model 148
99.60 %
Model 149
99.60 %
Model 150
99.61 %
Model 151
99.60 %
0
50
100
Satisfaction rate [%]

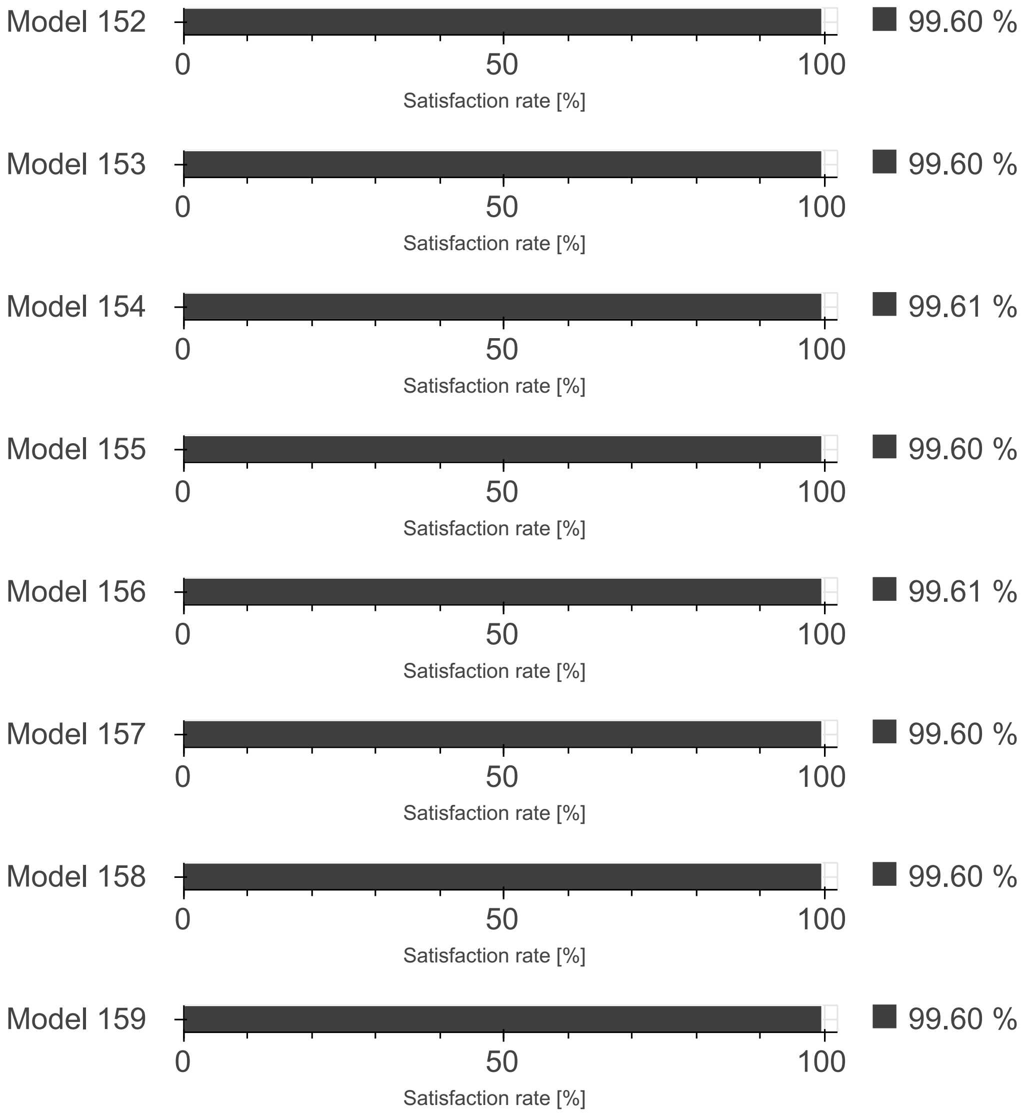

Data Quality

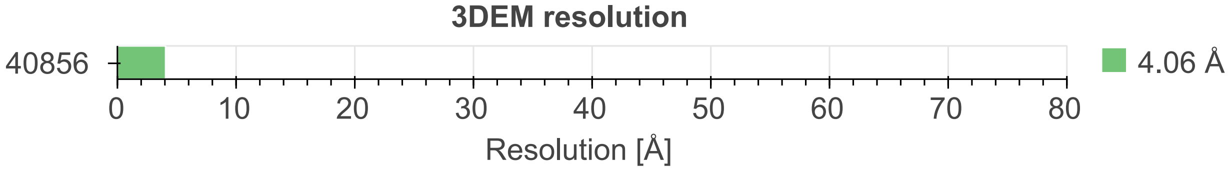

Fit to Data Used for Modeling

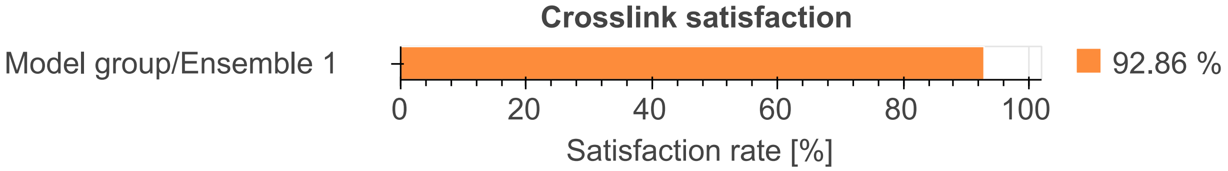

2. Model Details

2.1. Ensemble information

This entry consists of 1 distinct ensemble(s).

2.2. Representation

This entry has 1 representation(s).

ID	Model(s)	Entity ID	Molecule name	Chain(s) [auth]	Total residues	Rigid segments	Flexible segments	Model coverage/ Starting model coverage (%)	Scale
1	1-159	1	ORF2	A	1275	8-237, 250-258, 260-277, 284-310, 313-352, 353-359, 362-370, 375-381, 393-849, 857-862, 864-868, 873-955, 960-1030, 1033-1061, 1068-1275	1-7, 238-249, 259, 278-283, 311-312, 360-361, 371-374, 382-392, 850-856, 863, 869-872, 956-959, 1031-1032, 1062-1067	100.00 / 100.00	Coarse-grained: 1 residue(s) per bead

2.3. Datasets used for modeling

There are 16 unique datasets used to build the models in this entry.

ID	Dataset type	Database name	Data access code
1	De Novo model	AlphaFoldDB	AF-O00370-F1
2	De Novo model	MODEL ARCHIVE	ma-fejd6
3	De Novo model	MODEL ARCHIVE	ma-joo4d
4	De Novo model	MODEL ARCHIVE	ma-lzyrq
5	De Novo model	MODEL ARCHIVE	ma-xlzzy
6	Mass Spectrometry data	PRIDE	PXD038615
7	Crosslinking-MS data	Zenodo	10.5281/zenodo.10377421
8	Crosslinking-MS data	Zenodo	10.5281/zenodo.10377421
9	Crosslinking-MS data	Zenodo	10.5281/zenodo.10377421
10	EM raw micrographs	EMPIAR	EMPIAR-11556
11	3DEM volume	EMDB	40856
12	3DEM volume	Zenodo	10.5281/zenodo.10377421
13	De Novo model	MODEL ARCHIVE	ma-9wovj
14	2DEM class average	Zenodo	10.5281/zenodo.10377421
15	2DEM class average	Zenodo	10.5281/zenodo.10377421
16	2DEM class average	Zenodo	10.5281/zenodo.10377421

2.4. Methodology and software ?

This entry is a result of 1 distinct protocol(s).

Step number	Protocol ID	Method name	Method type	Method description	Number of computed models	Multi state modeling	Multi scale modeling
1	1	Sampling	AlphaFold2	Modeling of full-length ORF2p with AlphaFold2 using varying alignment depth. Details of the simulations are availabel in the ModelArchive entry ma-fejd6	Not available	False	False
2	1	Sampling	Molecular Dynamics simulations	Details of molecular dynamics simulations are available ModelArchive entries	Not available	False	False
3	1	Sampling	Replica Exchange Gibbs sampling, based on Metropolis Monte Carlo	20 replicas; 3 runs; 10000 models per run	30000	False	True
4	1	Refinement	Steepest descent	Conversion of a Ca-model to a full backbone model	159	False	False
5	1	Refinement	SCWRL	Conversion of a backbone model to afull-atom model	159	False	False
6	1	Refinement	Geometry optimization	Conversion of a backbone model to a full-atom model	159	False	False

There are 7 software packages reported in this entry.

ID	Software name	Software version	Software classification	Software location
7	Sampcon	2.18.0	validation	https://github.com/salilab/imp-sampcon
3	ColabFold	1.3.0	model building	https://github.com/sokrypton/ColabFold
4	GROMACS	2022.30	model building	https://www.gromacs.org/
1	IMP PMI module	2.19.0	integrative model building	https://integrativemodeling.org
5	PULCHRA	3.04	model building	https://sites.gatech.edu/cssb/pulchra/
6	SCWRL4.0	4.00	model building	http://dunbrack.fccc.edu/lab/scwrl
2	Integrative Modeling Platform (IMP)	2.19.0	integrative model building	https://integrativemodeling.org

3. Data quality ?

3.2. Crosslinking-MS

At the moment, data validation is only available for crosslinking-MS data deposited as a fully compliant dataset in the PRIDE Crosslinking database. Correspondence between crosslinking-MS and entry entities is established using pyHMMER. Only residue pairs that passed the reported threshold are used for the analysis. The values in the report have to be interpreted in the context of the experiment (i.e. only a minor fraction of in-situ or in-vivo dataset can be used for modeling).

Crosslinking-MS dataset is not available in the PRIDE Crosslinking database.

3.3. 3DEM

This section describes quality of the 3DEM datasets

40856

3.3.1. Experimental information

EM reconstruction method:	SINGLE PARTICLE
Resolution:	4.06 Å
Recommended level:	0.104
Estimated volume:	62.43 nm^3
Specimen preparation:	Preparation ID 1 Vitrification
Map-only validation report:	wwPDB validation report

3.3.2. Map visualisation

This section contains visualisations of the EMDB entry 40856. These allow visual inspection of the internal detail of the map and identification of artifacts. Images derived from a raw map, generated by summing the deposited half-maps, are presented below the corresponding image components of the primary map to allow further visual inspection and comparison with those of the primary map.

3.3.2.1. Orthogonal projections

Primary map

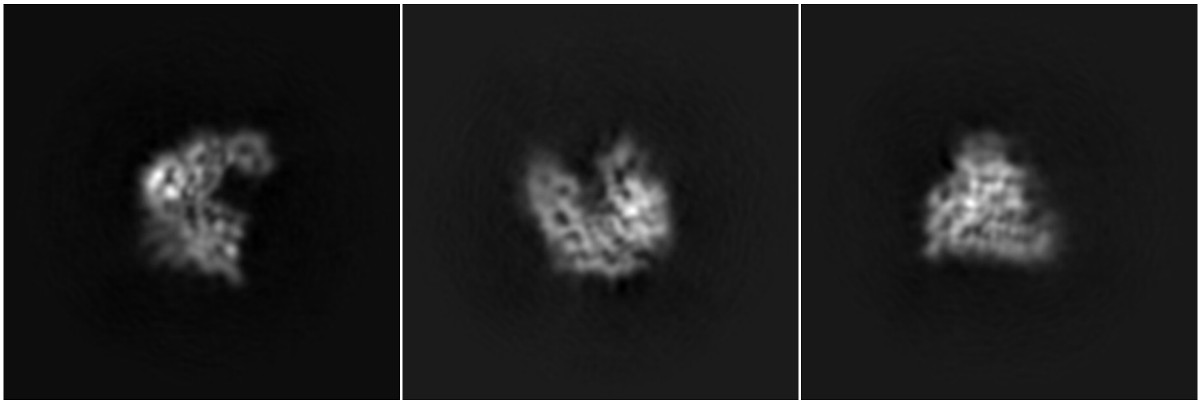

X **Y** **Z**

Raw map

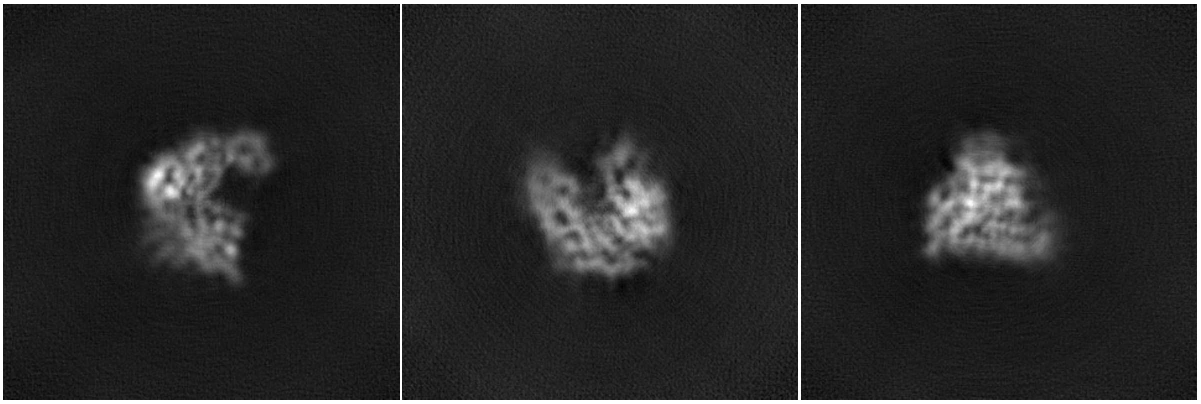

X Y Z

The images above show the map projected in three orthogonal directions.

3.3.2.2. Central slices ?

Primary map

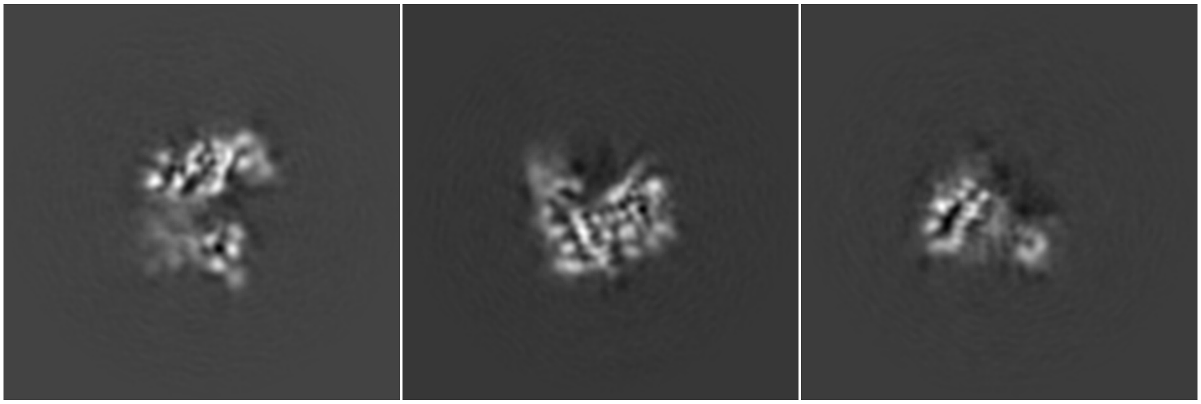

X Index: 160 Y Index: 160 Z Index: 160

Raw map

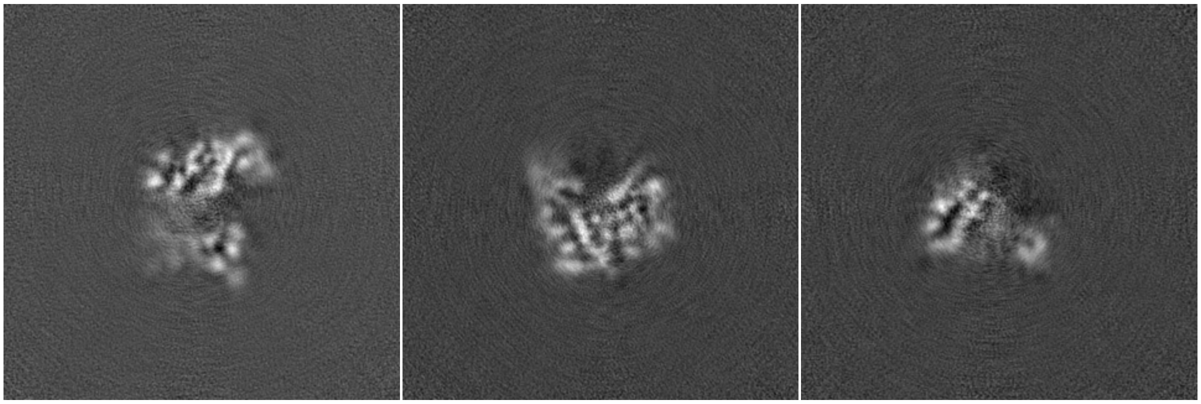

X Index: 160 Y Index: 160 Z Index: 160

The images above show central slices of the map in three orthogonal directions.

3.3.2.3. Largest variance slices ?

Primary map

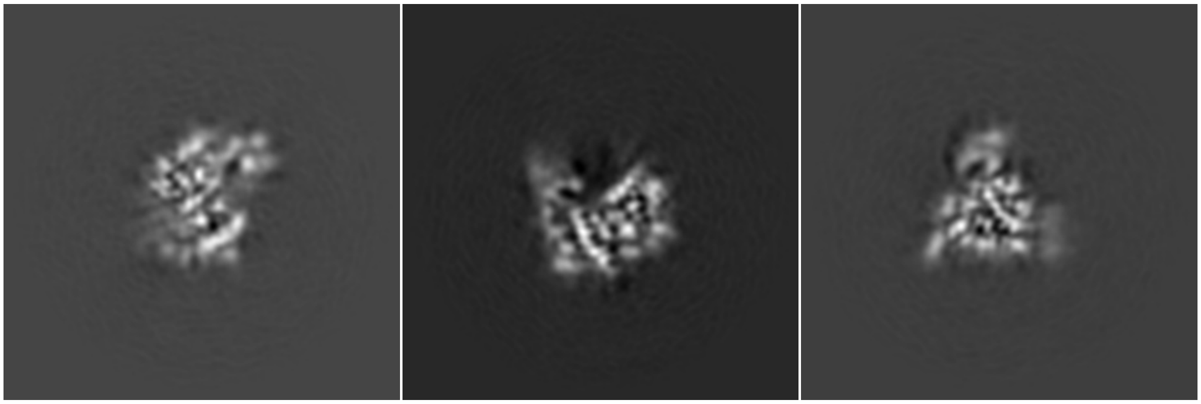

X Index: 137 **Y Index: 158** **Z Index: 185**

Raw map

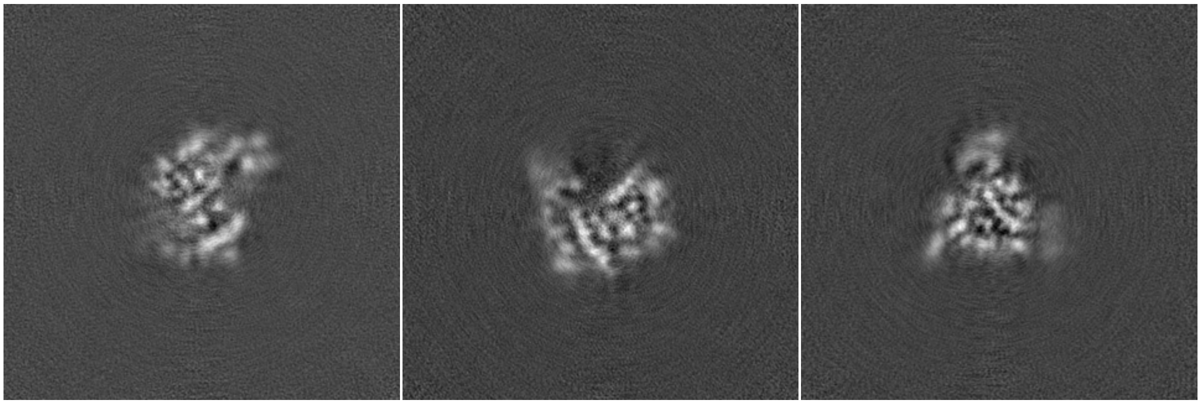

X Index: 137 **Y Index: 158** **Z Index: 185**

The images above show the largest variance slices of the map in three orthogonal directions.

3.3.2.4 Orthogonal standard-deviation projections (false-color) ?

Primary map

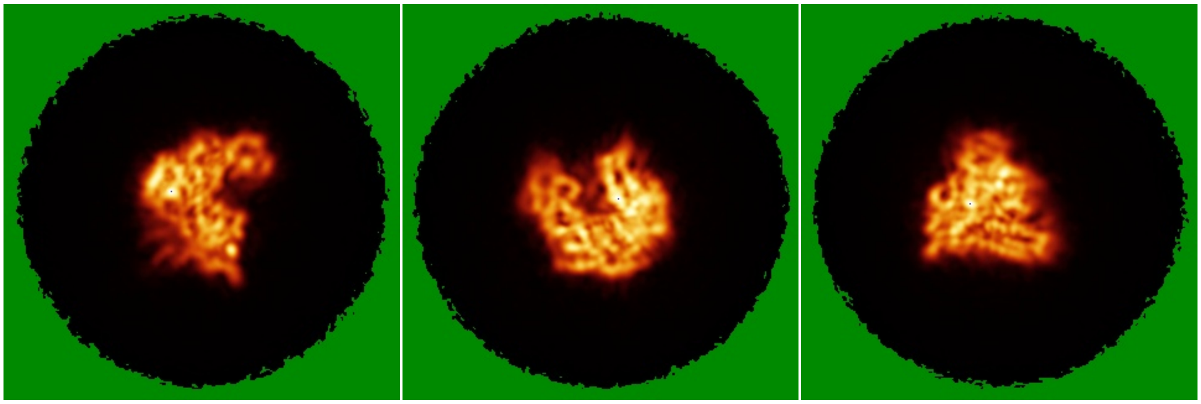

X **Y** **Z**

Raw map

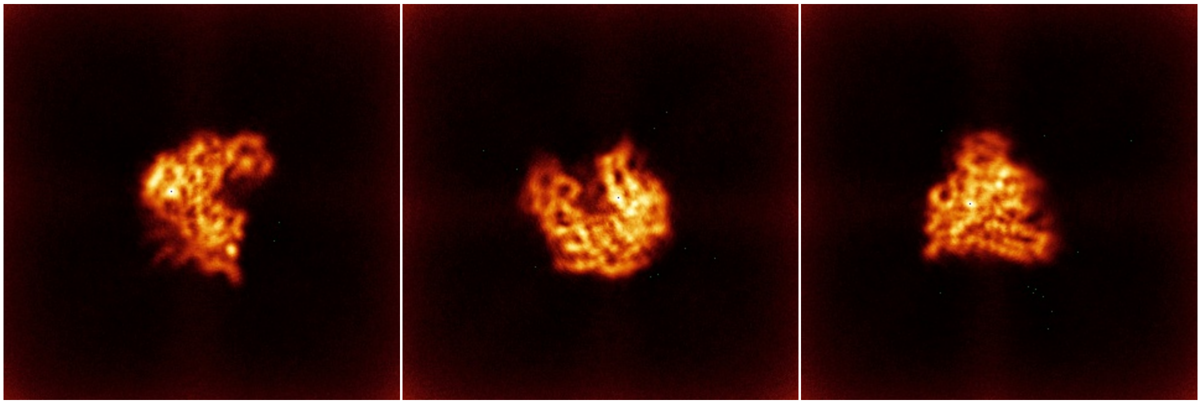

X **Y** **Z**

The images above show the map standard deviation projections with false color in three orthogonal directions. Minimum values are shown in green, max in blue, and dark to light orange shades represent small to large values respectively.

3.3.2.5. Orthogonal surface views ?

Primary map

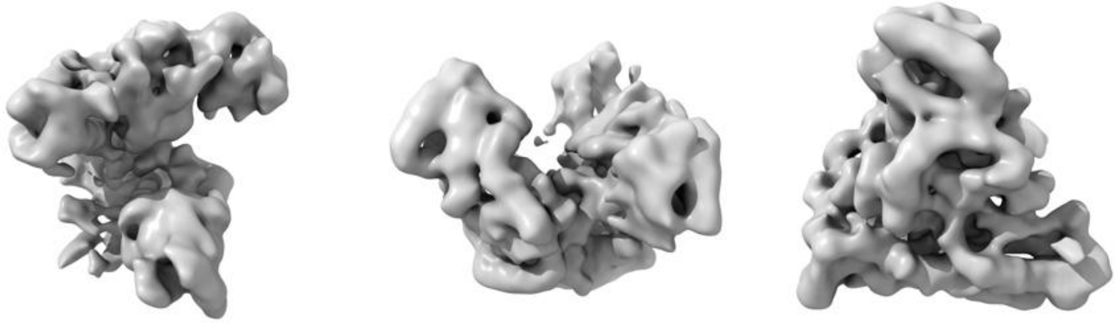

X **Y** **Z**

The images above show the 3D surface view of the map at the recommended contour level 0.104 . These images, in conjunction with the slice images, may facilitate assessment of whether an appropriate contour level has been provided.

Raw map

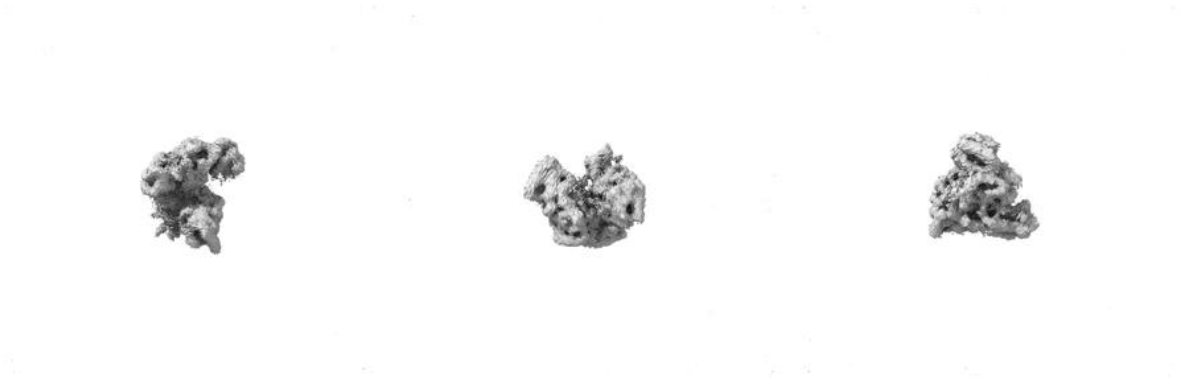

X **Y** **Z**

These images show the 3D surface of the raw map. The raw map’s contour level 0.070 was selected so that its surface encloses the same volume as the primary map does at its recommended contour level.

3.3.2.6. Mask visualisation ?

This section shows the 3D surface view of the primary map at 50% transparency overlaid with the specified mask at 0% transparency. A mask typically either:

- Encompasses the whole structure;
- Separates out a domain, a functional unit, a monomer or an area of interest from a larger structure.

emd_40856_msk_1.map ?

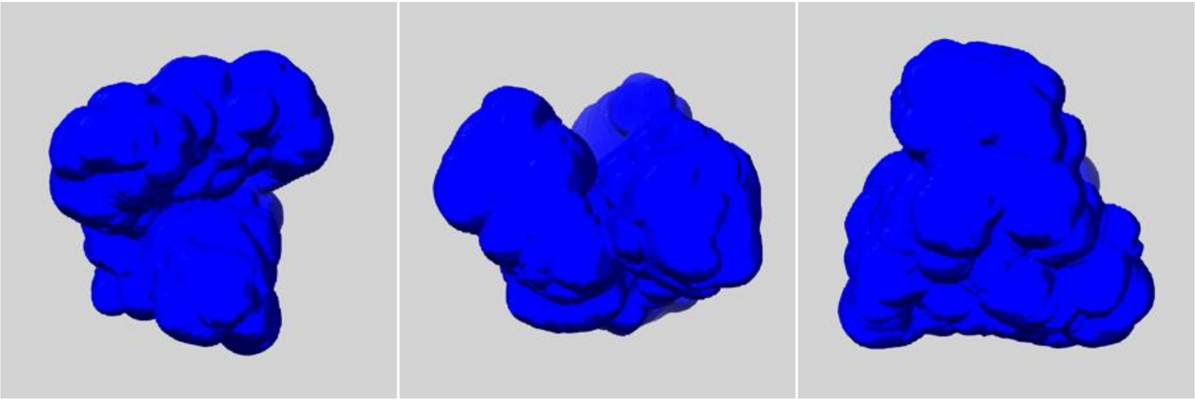

X **Y** **Z**

3.3.3. Map analysis ?

This section contains the results of statistical analysis of the map.

3.3.3.1. Map-value distribution ?

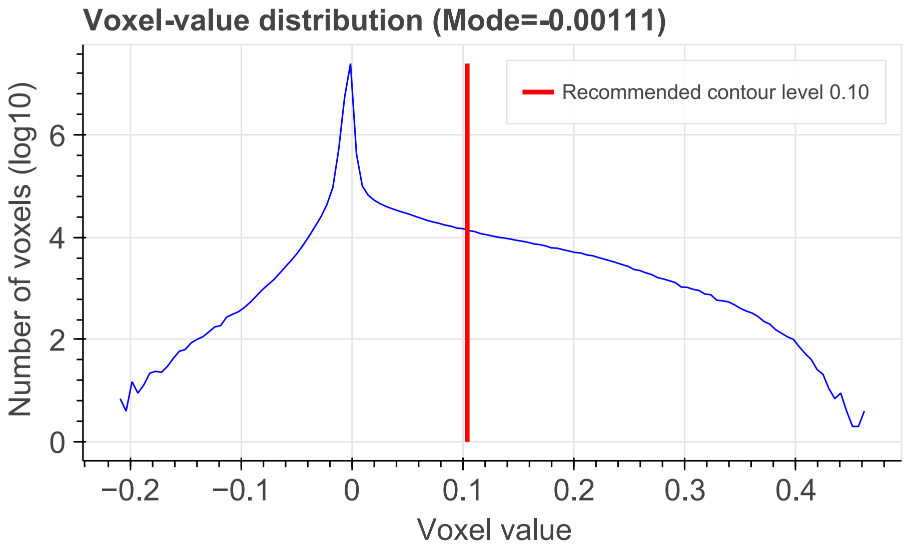

The map-value distribution is plotted in 128 intervals along the x-axis. The y-axis is logarithmic. A spike in this graph at zero usually indicates that the volume has been masked.

3.3.3.2. Volume estimate ?

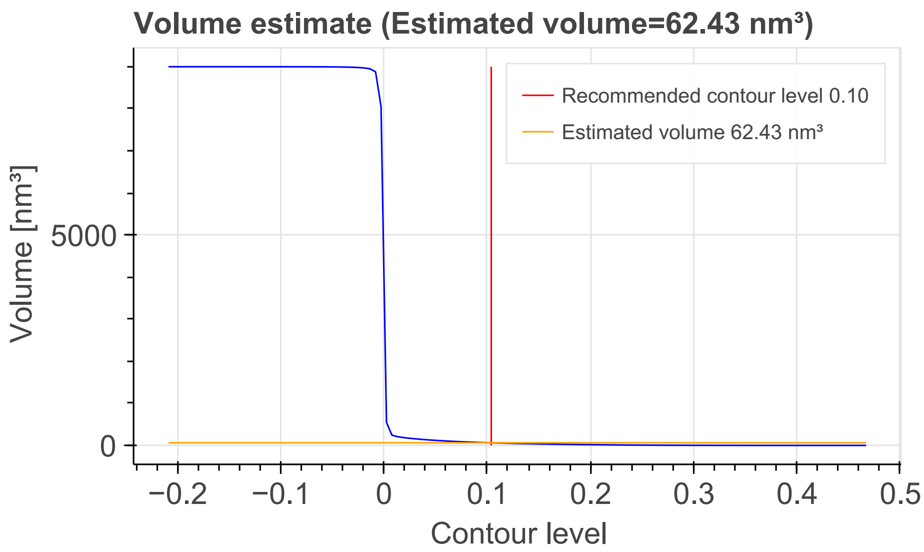

The volume at the recommended contour level is 62.43 nm³.

The volume estimate graph shows how the enclosed volume varies with the contour level. The recommended contour level is shown as a vertical line and the intersection between the line and the curve gives the volume of the enclosed surface at the given level.

3.3.3.3. Rotationally averaged power spectrum ?

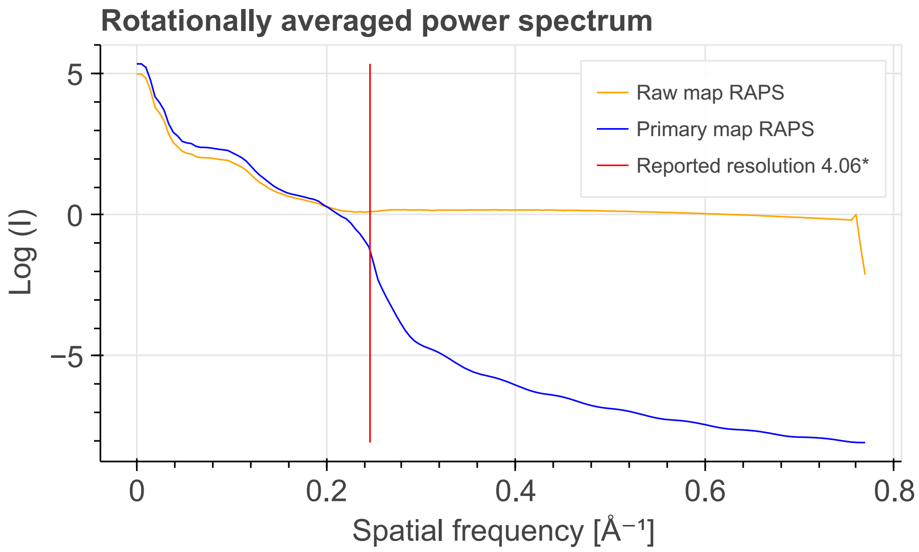

*Reported resolution corresponds to spatial frequency of 0.246 $Å^{-1}$

3.3.4. Fourier-Shell correlation ?

3.3.4.1. FSC ?

Fourier-Shell Correlation (FSC) is the most commonly used method to estimate the resolution of single-particle and subtomogram-averaged maps. The shape of the curve depends on the imposed symmetry, mask and whether or not the two 3D reconstructions used were processed from a common reference. The reported resolution is shown as a black line. A curve is displayed for the half-bit criterion in addition to lines showing the 0.143 gold standard cut-off and 0.5 cut-off.

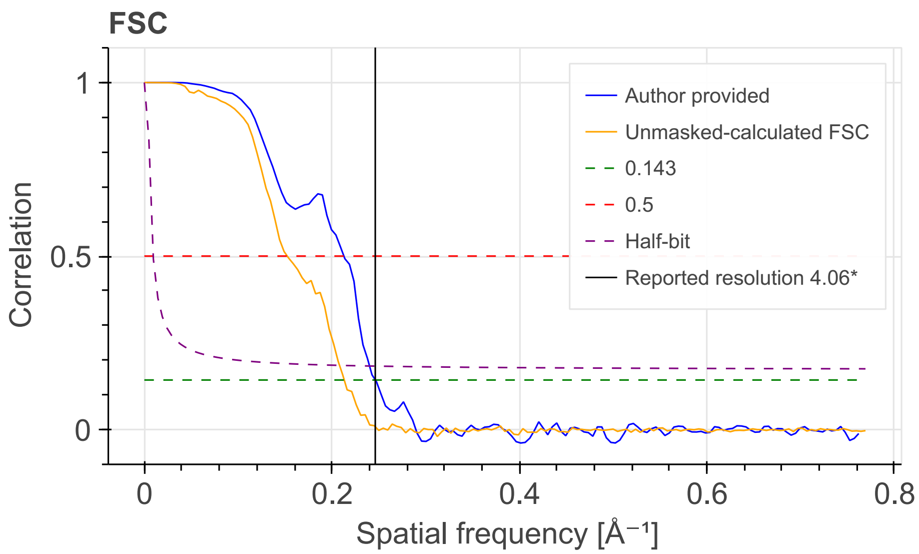

*Reported resolution corresponds to spatial frequency of 0.246 $Å^{-1}$

3.3.4.2. Resolution estimates ?

Resolution estimate (Å)	Estimation criterion (FSC cut-off)		
	**0.143**	**0.5**	**Half-bit**
Reported by author	4.06	-	-
Author-provided FSC curve	4.06	4.70	4.16
Unmasked-calculated*	4.69	6.56	4.80

*Resolution estimate based on FSC curve calculated by comparison of deposited half-maps. The value from deposited half-maps intersecting FSC 0.143 CUT-OFF 4.69 differs from the reported value 4.06 by more than 10%.

3.4. Mass Spectrometry ?

Validation for this section is under development.

3.4. 2DEM class average ?

Validation for this section is under development.

3.4. EM raw micrographs ?

Validation for this section is under development.

4. Model quality ?

For models with atomic structures, MolProbity analysis is performed. For models with coarse-grained or multi-scale structures, excluded volume analysis is performed.

4.1a. Excluded Volume Analysis ?

Excluded volume satisfaction for the models in the entry are listed below. The Analysed column shows the number of particle-partice or particle-atom pairs for which excluded volume was analysed.

Model ID	Analysed	Number of violations	Excluded Volume Satisfaction (%)
1	812175	3213	99.60
2	812175	3209	99.60
3	812175	3214	99.60
4	812175	3213	99.60
5	812175	3208	99.61
6	812175	3218	99.60
7	812175	3219	99.60
8	812175	3225	99.60
9	812175	3219	99.60
10	812175	3207	99.61
11	812175	3218	99.60
12	812175	3214	99.60
13	812175	3207	99.61
14	812175	3215	99.60
15	812175	3217	99.60
16	812175	3210	99.60
17	812175	3215	99.60
18	812175	3214	99.60
19	812175	3207	99.61
20	812175	3208	99.61
21	812175	3212	99.60
22	812175	3214	99.60
23	812175	3215	99.60
24	812175	3211	99.60
25	812175	3208	99.61
26	812175	3201	99.61
27	812175	3212	99.60
28	812175	3203	99.61
29	812175	3207	99.61
30	812175	3220	99.60
31	812175	3214	99.60
32	812175	3216	99.60
33	812175	3218	99.60
34	812175	3213	99.60
35	812175	3207	99.61

Model ID	Analysed	Number of violations	Excluded Volume Satisfaction (%)
36	812175	3217	99.60
37	812175	3219	99.60
38	812175	3215	99.60
39	812175	3211	99.60
40	812175	3212	99.60
41	812175	3206	99.61
42	812175	3221	99.60
43	812175	3214	99.60
44	812175	3211	99.60
45	812175	3218	99.60
46	812175	3218	99.60
47	812175	3218	99.60
48	812175	3212	99.60
49	812175	3220	99.60
50	812175	3202	99.61
51	812175	3206	99.61
52	812175	3219	99.60
53	812175	3205	99.61
54	812175	3218	99.60
55	812175	3208	99.61
56	812175	3211	99.60
57	812175	3214	99.60
58	812175	3202	99.61
59	812175	3210	99.60
60	812175	3203	99.61
61	812175	3209	99.60
62	812175	3221	99.60
63	812175	3220	99.60
64	812175	3220	99.60
65	812175	3209	99.60
66	812175	3213	99.60
67	812175	3212	99.60
68	812175	3212	99.60
69	812175	3207	99.61
70	812175	3202	99.61

Model ID	Analysed	Number of violations	Excluded Volume Satisfaction (%)
71	812175	3214	99.60
72	812175	3217	99.60
73	812175	3215	99.60
74	812175	3207	99.61
75	812175	3207	99.61
76	812175	3210	99.60
77	812175	3219	99.60
78	812175	3214	99.60
79	812175	3215	99.60
80	812175	3204	99.61
81	812175	3212	99.60
82	812175	3220	99.60
83	812175	3215	99.60
84	812175	3209	99.60
85	812175	3209	99.60
86	812175	3214	99.60
87	812175	3208	99.61
88	812175	3215	99.60
89	812175	3215	99.60
90	812175	3218	99.60
91	812175	3207	99.61
92	812175	3202	99.61
93	812175	3213	99.60
94	812175	3214	99.60
95	812175	3216	99.60
96	812175	3206	99.61
97	812175	3218	99.60
98	812175	3207	99.61
99	812175	3208	99.61
100	812175	3211	99.60
101	812175	3210	99.60
102	812175	3217	99.60
103	812175	3211	99.60
104	812175	3220	99.60
105	812175	3205	99.61

Model ID	Analysed	Number of violations	Excluded Volume Satisfaction (%)
106	812175	3215	99.60
107	812175	3216	99.60
108	812175	3219	99.60
109	812175	3210	99.60
110	812175	3213	99.60
111	812175	3212	99.60
112	812175	3197	99.61
113	812175	3212	99.60
114	812175	3214	99.60
115	812175	3224	99.60
116	812175	3214	99.60
117	812175	3207	99.61
118	812175	3211	99.60
119	812175	3209	99.60
120	812175	3211	99.60
121	812175	3210	99.60
122	812175	3206	99.61
123	812175	3216	99.60
124	812175	3216	99.60
125	812175	3213	99.60
126	812175	3221	99.60
127	812175	3211	99.60
128	812175	3212	99.60
129	812175	3218	99.60
130	812175	3207	99.61
131	812175	3217	99.60
132	812175	3213	99.60
133	812175	3207	99.61
134	812175	3217	99.60
135	812175	3214	99.60
136	812175	3217	99.60
137	812175	3219	99.60
138	812175	3206	99.61
139	812175	3205	99.61
140	812175	3214	99.60

Model ID	Analysed	Number of violations	Excluded Volume Satisfaction (%)
141	812175	3214	99.60
142	812175	3209	99.60
143	812175	3211	99.60
144	812175	3205	99.61
145	812175	3219	99.60
146	812175	3228	99.60
147	812175	3218	99.60
148	812175	3214	99.60
149	812175	3213	99.60
150	812175	3203	99.61
151	812175	3216	99.60
152	812175	3216	99.60
153	812175	3214	99.60
154	812175	3207	99.61
155	812175	3216	99.60
156	812175	3206	99.61
157	812175	3217	99.60
158	812175	3217	99.60
159	812175	3217	99.60

4.2. PrISM Precision Analysis

*Regions of **low** **high** precision, defined as the variability among the models that satisfy the input data and calculated as the density-weighted root mean-square fluctuation (RMSF) from the bead/atom center of density, annotated and visualized using PrISM. The per-bead precision is computed from the deposited ensemble of superposed integrative models. High- and low-precision regions are then determined by clustering beads of similar precision based on their proximity in the structure. Only coarse-grained beads (or CA atoms for atomic models) of deposited models are used for assessment and visualization, and three projections for each representative model are generated.*

PrISM analysis for Ensemble 1 (models deposited/total: 159/1383).

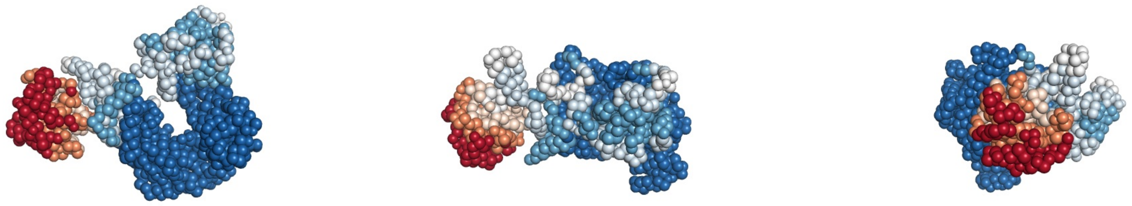

5. Fit to Data Used for Modeling Assessment

5.2. Crosslinking-MS

5.2.1. Restraint types

This table summarizes information about crosslinker(s) used for data generation, and how crosslinking information was translated into actual modeling restraints. Restraints assigned "by-residue" are interpreted as between CA atoms. Restraints between coarse-grained beads are indicated as "coarse-grained". Restraint group represents a set of crosslinking restraints applied collectively in the modeling.

There are 56 crosslinking restraints combined in 56 restraint groups.

Linker	Residue 1	Atom 1	Residue 2	Atom 2	Restraint type	Distance, Å	Count
BS3	LYS	CA	LYS	CA	upper bound	27.00	56

Distograms of individual restraints

Distograms (i.e., histogram plots of distances) provide an overview of distributions of distances between residues for which chemical crosslinks were identified. The shift of the distogram relative to the threshold value may indicate a poor model. Restraints with identical thresholds are grouped into one plot. Only the best distance per restraint per model group/ensemble is plotted. Inter- and intramolecular (including self-links) restraints are also grouped into one plot. Distance for a restraint between coarse-grained beads is calculated as a minimal distance between shells; if beads intersect, the distance will be reported as 0.0. A bead with the highest available resolution for a given residue is used for the assessment.

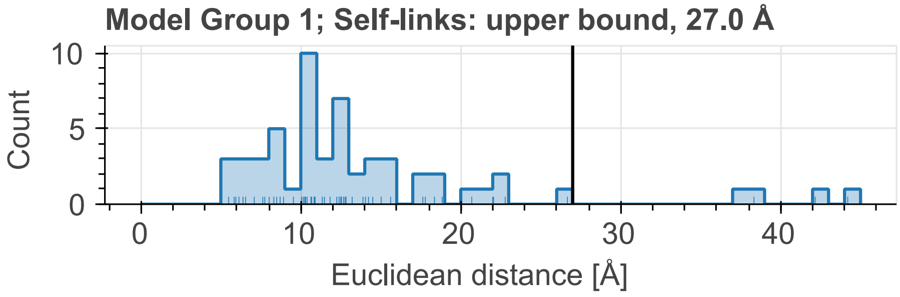

5.2.2. Satisfaction of restraints

Satisfaction of restraints is calculated on a restraint group (a set of crosslinking restraints applied collectively in the modeling) level. Satisfaction of a restraint group depends on satisfaction of individual restraints in the group and the conditionality (all/any). A restraint group is considered satisfied, if the condition was met in at least one model of the model group/ensemble. The number of measured restraints can be smaller than the total number of restraint groups if crosslinks involve non-modeled residues. Only deposited models are used for validation right now.

State group	State	Model group	# of Deposited models/Total	Restraint group type	Satisfied (%)	Violated (%)	Count (Total=56)
1	1	1	159/1383	All	92.86	7.14	56
				Self-links/ Intramolecular	92.86	7.14	56

Per-model satisfaction rates in ensembles

Every point represents one model in a model group/ensemble. Where possible, boxplots with quartile marks are also plotted.

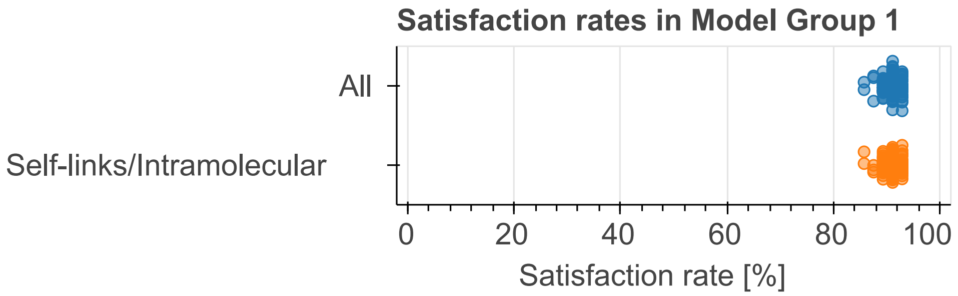

5.3. 3DEM

This section describes fit of models to the 3DEM data. Only results for the representative model, selected as a first model with the largest number of asymmetric units.

3DEM validation for coarse-grained structures is under development.

5.4. Mass Spectrometry ?

Validation for this section is under development.

5.4. 2DEM class average ?

Validation for this section is under development.

5.4. EM raw micrographs ?

Validation for this section is under development.

6. Fit to Data Used for Validation Assessment ?

Validation for this section is under development.

Acknowledgments

The development of integrative model validation metrics, implementation of a model validation pipeline, and creation of a validation report for integrative structures are funded by NSF awards to the PDB-IHM team (DBI-1756248, DBI-2112966, DBI-2112967, DBI-2112968, and DBI-1756250) and awards from NSF, NIH, and DOE to the RCSB PDB (DBI-2321666, R01GM157729, and DE-SC0019749). The PDB-IHM team and members of the Sali lab contributed model validation metrics and software packages.

Dr. Jill Trewhella, Dr. Dina Schneidman, and members of the SASBDB repository are acknowledged for their advice and support in implementing SAS validation methods. Team members from the labs of Dr. Juri Rappsilber, Dr. Alexander Leitner, Dr. Andrea Graziadei, and members of PRIDE database are acknowledged for their advice and support in implementing crosslinking-MS validation methods. We are grateful to Dr. Shruthi Viswanath for discussions about uncertainty assessment of integrative structural models.

Members of the wwPDB Integrative/Hybrid Methods Task Force provided recommendations and community support for the project.
